# Event-Driven Multimodal Sensing and Computing for Context-Aware Home Monitoring Using Stereo Vision and Dietary Event Anchoring

**DOI:** 10.3390/s26154803

**Published:** 2026-07-28

**Authors:** Zhaozhen Tong, Kumiko Ono, Masahide Nakamura, Sinan Chen

**Affiliations:** 1 Graduate School of Engineering, Faculty of Engineering, Kobe University, 1-1 Rokkodai-cho, Nada, Kobe 657-8501, Japan; 243t804t@gsuite.kobe-u.ac.jp (Z.T.); masa-n@cmds.kobe-u.ac.jp (M.N.); 2 Graduate School of Health Sciences, Kobe University, 7-10-2 Tomogaoka, Suma, Kobe 654-0142, Japan; onoku@tiger.kobe-u.ac.jp; 3 Center of Mathematical and Data Sciences, Kobe University, 1-1 Rokkodai-cho, Nada, Kobe 657-8501, Japan

**Keywords:** multimodal sensing, event-driven monitoring, context-aware computing, home monitoring, stereo vision, human motion sensing, dietary event anchoring, smart healthcare

## Abstract

Real-world home monitoring requires sensing systems that can capture daily behaviour without continuous raw-video retention or excessive user burden. However, domestic environments present irregular activity timing, fragmented human presence, asynchronous multimodal events, and privacy-sensitive data management. This study proposes an event-driven multimodal sensing and computing framework for context-aware home monitoring using stereo vision and dietary event anchoring. The framework integrates stereo RGB-based three-dimensional human motion sensing, dining-zone-triggered meal image acquisition, runtime event orchestration, timestamp-based cross-modal synchronization, privacy-aware local storage, and large-language-model-assisted dietary context interpretation. Instead of continuously recording all sensor streams, the system activates and organizes sensing through human presence detection, debounce logic, cooldown-based session control, and dining-zone occupancy events. Meal-related events are used as contextual anchors to associate motion sessions and dietary observations into synchronized behavioural episodes. The prototype was deployed for 11 consecutive days in a real kitchen–dining environment, with the stabilized real-time monitoring phase evaluated from 11 to 14 February 2026. During this phase, the system generated 26 event-driven motion sessions and 51,165 captured pose frames, of which 25,925 were valid. Sustained active sessions accounted for 30.8% of all sessions but contributed 81.5% of captured pose frames, indicating that event-driven orchestration concentrated motion data within behaviourally meaningful activity windows. Eight meal-related records were obtained, seven of which overlapped with motion sessions, resulting in 87.5% meal-event overlap coverage. Structured pose outputs required approximately 550 kB/min, corresponding to about 33 MB/h of recorded pose data. LLM-assisted meal-image interpretation achieved a mean absolute percentage error of 25.44%, supporting its use for coarse dietary-context description rather than precise nutritional quantification. However, this result is interpreted only as evidence for coarse dietary-context description and not as validation of a precise nutritional or clinical dietary assessment method. These results demonstrate the system-level feasibility of transforming irregular domestic observations into structured, temporally indexed, and privacy-aware multimodal behavioural records for future home monitoring applications.

## 1. Introduction

Population aging and the increasing need for long-term care have accelerated the development of home-based sensing systems that can support independent living, daily health management, and the early detection of functional change. Smart home and ambient assisted living technologies have been investigated as practical tools for monitoring activities of daily living, supporting chronic disease management, and reducing the burden on caregivers [1,2,3,4,5]. Recent reviews further indicate that sensor technologies for older adults have evolved from simple detection devices toward intelligent systems that combine environmental sensing, wearable sensing, Internet of Things (IoT) infrastructure, and artificial intelligence for personalized and proactive monitoring [1,2]. However, translating these technologies into real homes remains challenging because domestic behaviour is irregular, context-dependent, and embedded in private living spaces.

A major requirement for home monitoring is the ability to observe daily behaviour without imposing excessive burden on users. Wearable devices can capture physiological and inertial signals but require wearing compliance, charging, and user acceptance. Environmental sensors are less intrusive but may provide only indirect information about movement. Camera-based sensing can capture rich movement information without requiring body attachment, and markerless motion capture has recently shown increasing potential for clinical and functional movement analysis [6,7,8,9]. Nevertheless, vision-based systems raise privacy concerns and may be affected by occlusion, lighting, camera placement, and uncontrolled daily-life activity. These issues are especially important in home environments, where continuous raw-video recording is often difficult to justify. The acceptance of smart home and video-based monitoring systems depends not only on technical accuracy but also on privacy, autonomy, transparency, and user control. Recent studies have shown that older adults and caregivers may recognize the potential value of smart home technologies, while still expressing concerns about surveillance, data access, loss of privacy, and changes to normal routines [10,11,12,13]. Privacy-aware camera-based monitoring and subject anonymization have therefore become active research topics [14]. These findings suggest that future home-monitoring systems should reduce unnecessary visual data retention, organize sensing around meaningful events, and provide structured outputs that are useful for analysis without requiring continuous video archives. Another important direction is the construction of digital behavioural biomarkers from daily-life data. Digital biomarkers derived from sensors and smart home technologies may capture behavioural, physiological, and environmental indicators that are difficult to measure through occasional clinical assessments alone [15,16]. For example, changes in movement continuity, activity fragmentation, daily routine regularity, and behaviour around specific domestic contexts may provide useful signals for future functional or cognitive assessment. However, the development of such biomarkers requires structured, longitudinal, and context-aware records rather than isolated sensor frames or manually segmented laboratory trials.

Dietary behaviour is one such meaningful daily-life context. Meal-related routines involve approaching the dining area, sitting, reaching, standing, handling tableware, and post-meal movement. At the same time, dietary intake and meal context are important for chronic disease management, nutrition care, and lifestyle monitoring. AI-based dietary assessment and image-based food analysis have advanced rapidly in recent years [17,18,19]. However, most dietary-image studies focus on food recognition, portion estimation, or nutrient estimation as independent tasks. In contrast, for home monitoring, meal-related events can also serve as contextual anchors for interpreting surrounding movement behaviour. This perspective requires integration between dietary-event sensing and motion sensing. Despite these developments, three gaps remain. First, many home-monitoring studies focus on activity recognition, sensor integration, or clinical feasibility, but less attention is given to runtime orchestration: when the system should activate sensing, how should activity sessions be bounded and how should fragmented presence be handled? Second, vision-based motion monitoring and image-based dietary assessment are usually developed separately, although daily movement and meal-related activity are closely connected in home routines. Third, privacy-aware monitoring requires not only anonymization or access control, but also data minimization and structured local storage that avoid unnecessary retention of continuous raw videos.

To address these gaps, this study proposes an event-driven multimodal sensing and computing framework for context-aware home monitoring using stereo vision and dietary event anchoring. The proposed framework is designed not as a new pose estimation algorithm or a standalone dietary assessment method, but as a deployable sensing–computing architecture that coordinates multimodal data acquisition, runtime event control, cross-modal temporal organization, and privacy-aware local data representation. Specifically, stereo RGB-based motion sensing is used to generate three-dimensional skeletal trajectories, while dining-zone-triggered image acquisition provides meal-related contextual events. These heterogeneous observations are organized through event-driven session control and timestamp-based synchronization, enabling irregular domestic activities to be transformed into structured session-level and episode-level behavioural records.

In response to these challenges, the present study is positioned as a systems-oriented feasibility study. The goal is not to propose a new pose-estimation algorithm, a clinically validated dietary assessment method, or a diagnostic model. Instead, the focus is on designing and evaluating a deployable sensing and computing framework that can organize irregular home observations into structured multimodal behavioural records. This positioning is important because the practical value of home monitoring depends not only on sensing accuracy, but also on event triggering, temporal organization, privacy-aware storage, and the ability to link heterogeneous observations into interpretable episodes. The main contributions of this study are as follows:

1. We propose a multimodal sensing and computing framework for real-world home monitoring that integrates stereo vision-based human motion sensing, dining-zone-triggered dietary event acquisition, and local edge-side sensing, event orchestration, pose processing, and structured data storage within a unified prototype system.
2. We design an event-driven runtime orchestration mechanism based on human presence detection, debounce logic, and cooldown-based session control to activate sensing, maintain session continuity, and convert fragmented domestic activity into bounded motion sessions.
3. We introduce meal-event anchoring and timestamp-based cross-modal synchronization to associate asynchronous motion sessions with dietary events and construct synchronized behavioural episodes for context-aware monitoring.
4. We implement a privacy-aware structured data representation that stores skeletal trajectories, timestamps, session metadata, selected meal images, dietary-context descriptors, and episode records rather than relying on continuous raw-video retention.
5. We evaluate the prototype as a system-level feasibility study in a real kitchen–dining environment, using quantitative indicators related to session generation, pose-data usability, motion–meal overlap, storage cost, and coarse dietary-context interpretation.

The remainder of this paper is organized as follows. Section 2 reviews related work on vision-based motion monitoring, multimodal sensing for healthcare and smart homes, event-driven and context-aware sensing, and dietary context monitoring. Section 3 describes the proposed framework, including sensing components, runtime orchestration, dining-zone detection, temporal synchronization, data representation, and LLM-assisted dietary interpretation. Section 4 presents the deployment environment, evaluation metrics, and experimental results. Section 5 discusses the implications, limitations, privacy considerations, scalability, and future directions. Finally, Section 6 concludes the paper.

## 2. Related Work

### 2.1. Vision-Based Human Motion and Activity Monitoring

Vision-based human motion and activity monitoring has become an important research direction for healthcare, assisted living, smart homes, and human–computer interaction. Compared to wearable sensors, camera-based systems can capture movement without requiring the user to wear, charge, or maintain sensing devices. This advantage is particularly relevant for home monitoring applications involving older adults or people who may have difficulty operating wearable devices continuously. Recent surveys have shown that vision-based human action recognition has evolved from handcrafted appearance and motion descriptors to deep learning-based representations using RGB frames, optical flow, depth information, and skeletal keypoints [20,21].

Human pose estimation is one of the key enabling technologies for vision-based motion monitoring. Deep learning-based pose estimation methods infer two-dimensional or three-dimensional body keypoints from images and videos, making it possible to represent human motion as structured skeletal trajectories rather than raw image sequences. Recent pose estimation surveys have summarized major advances in 2D and 3D human pose estimation, including top-down and bottom-up pipelines, monocular and multi-view settings, temporal modelling, and robustness challenges such as occlusion, depth ambiguity, and limited generalization across environments [22,23,24]. These developments provide the technical basis for extracting motion information from camera streams in a more compact and analysis-ready form. Skeleton-based representations are especially attractive for healthcare-oriented monitoring because they reduce the dependence on appearance information and enable quantitative analysis of posture and movement. In skeleton-based action recognition, human motion is usually represented as a sequence of joint coordinates, and learning models such as recurrent networks, graph convolutional networks, and transformers are used to capture spatial–temporal relationships among body joints [25]. Compared to raw RGB videos, skeletal representations can reduce sensitivity to background appearance, clothing, and scene texture. They also provide a more privacy-aware intermediate representation because they preserve motion structure while reducing the amount of identifiable visual information retained from the environment.

Recent work has also explored vision-based systems for functional movement and gait-related assessment. For example, smartphone- or camera-based approaches have been investigated for estimating gait speed, knee flexion, and other movement-related indicators that are relevant to mobility and fall-risk assessment [26]. Transfer learning and deep learning have further improved the ability of vision-based models to adapt to different activity-recognition tasks and limited datasets [27]. These studies indicate that vision-based motion features can support quantitative assessment of human movement and may complement conventional clinical or laboratory measurements. Despite these advances, much of the existing literature still focuses on algorithmic recognition accuracy, benchmark datasets, or controlled assessment protocols. In many studies, activities are predefined, recording windows are manually segmented, and the environment is relatively constrained. Such settings are valuable for validating pose estimation or activity classification models, but they do not fully address the practical requirements of real home monitoring. In domestic environments, activity timing is irregular, human presence is fragmented, occlusion is common, and meaningful behavioural information may appear only during specific daily-life contexts. Therefore, a deployable home monitoring system must not only estimate pose or classify activity, but also decide when to activate sensing, how to segment activity into usable sessions, and how to organize the resulting records for later analysis.

This gap is also evident when comparing laboratory-oriented motion sensing with passive in-home monitoring. Prior work has substantially advanced the representational accuracy of vision-based gait analysis and activity recognition. However, runtime orchestration, event-driven session control, and context-aware data organization have received less attention as first-class system design problems. The previous version of the present study also identified this gap, noting that existing vision-based gait and functional assessment studies often emphasize sensing accuracy under controlled conditions, whereas deployable home monitoring requires adaptation to irregular activity timing, fragmented presence, asynchronous events, and resource-aware execution. The present study builds on this line of research but shifts the emphasis from activity classification alone to event-driven multimodal sensing and computing. Instead of treating camera streams as continuous videos to be classified, the proposed framework uses stereo vision-based pose reconstruction, presence-triggered session control, dining-event anchoring, timestamp-based synchronization, and structured local storage. In this sense, the contribution is not a new pose estimation algorithm, but a system-level framework for transforming irregular domestic observations into structured behavioural records that can support context-aware home monitoring.

### 2.2. Multimodal Sensing for Healthcare and Smart Home Monitoring

Multimodal sensing has become a central direction in healthcare and smart home monitoring because daily health-related behaviour cannot be fully understood from a single data stream. Smart home health monitoring systems commonly integrate heterogeneous sensing sources, such as cameras, environmental sensors, wearable devices, ambient sensors, physiological sensors, microphones, smart appliances, and contextual logs. Recent reviews of smart home technologies for health monitoring have shown that such systems are increasingly used to support remote monitoring, aging in place, chronic disease management, independent living, and early detection of behavioural change [28,29]. These studies indicate that the home is becoming an important site for continuous and unobtrusive health-related data collection.

In ambient assisted living and in-home monitoring, multimodal sensing is often motivated by the limitations of single-modality systems. A single camera may capture posture and movement but may be sensitive to occlusion, illumination, and field-of-view constraints. Wearable devices can provide physiological or inertial signals but require user compliance. Environmental sensors can detect object use or room-level activity but often lack detailed motion information. Therefore, multimodal systems combine complementary modalities to improve coverage, robustness, and contextual interpretation. For example, Ghorbani et al. proposed a decision-aware ambient assisted living system using IoT embedded devices for in-home monitoring of older adults, demonstrating how sensing, decision logic, and user feedback can be integrated into an assisted-living architecture [30]. Recent surveys on multimodal information fusion for smart healthcare further emphasize that the value of multimodal sensing lies not only in collecting heterogeneous data, but also in transforming data into information, knowledge, and decision support [31]. In healthcare applications, multimodal fusion may occur at the signal level, feature level, decision level, or semantic level. Each fusion strategy has different implications for system design. Signal-level fusion requires precise synchronization and compatible sampling structures, whereas semantic or event-level fusion can support more flexible integration of asynchronous observations. This distinction is particularly relevant for home monitoring, where sensor streams may be activated at different times and may not always provide continuous parallel data.

Contactless health monitoring has also received increasing attention in recent years. Vision-based cameras, radar sensors, thermal imaging, and ambient sensing can measure or infer health-related information without requiring physical contact with the user. Hassanpour and Yang reviewed contactless vital sign monitoring with an emphasis on multimodal sensing and multi-task learning, highlighting the potential of combining complementary sensing technologies while also noting challenges related to environmental robustness, sensor fusion complexity, privacy, and clinical validation [32]. Although the present study focuses on body motion and dietary context rather than vital signs, the same principle applies: multimodal sensing can provide richer context than a single sensor, but it requires careful orchestration, synchronization, and interpretation. Smart home monitoring is also increasingly discussed in relation to digital twins and sensor-enabled smart environments. Sensor-enabled digital twins aim to construct dynamic representations of individuals, environments, or healthcare processes by integrating real-time sensor data, contextual information, and computational models [33]. This perspective is closely related to long-term home monitoring because behavioural records collected from daily life may provide the data foundation for future personalized models. However, digital twin-oriented systems require structured, temporally indexed, and semantically meaningful data. Raw sensor streams alone are insufficient unless they are organized into interpretable records that can support downstream modelling and decision-making.

Despite this progress, several limitations remain in existing multimodal smart home monitoring research. First, many systems emphasize sensor integration or classification performance but provide less detail on runtime event orchestration. In real homes, meaningful events occur irregularly, and continuous recording may be inefficient or privacy-sensitive. Second, multimodal systems often assume that different sensors can be synchronized continuously, whereas domestic monitoring may require flexible event-level association between asynchronous streams. Third, contextual information such as meal-related activity is often treated separately from motion monitoring, even though daily routines such as dining can provide meaningful anchors for interpreting movement. Fourth, privacy-aware data minimization and structured local storage are not always considered as core architectural elements. The present study addresses these gaps by proposing an event-driven multimodal sensing and computing framework for home monitoring. The framework integrates stereo vision-based motion sensing, dining-zone-triggered dietary image acquisition, timestamp-based cross-modal synchronization, structured local storage, and LLM-assisted dietary context interpretation. Compared to conventional multimodal monitoring systems that focus mainly on sensor fusion or activity classification, the proposed framework emphasizes how heterogeneous domestic observations can be activated, bounded, synchronized, and represented as meal-anchored behavioural episodes. This design is intended to support practical and privacy-aware monitoring in real home environments.

### 2.3. Event-Driven and Context-Aware Sensing Systems

Event-driven and context-aware sensing systems are increasingly important for smart home monitoring because domestic activities are not performed as isolated, pre-segmented tasks. In real homes, meaningful behaviours occur intermittently, are influenced by spatial context, and may involve multiple short transitions between rooms, furniture, objects, and activity zones. Therefore, a sensing system must not only recognize human activity, but also decide when data should be acquired, how event boundaries should be defined, and how heterogeneous observations should be interpreted in relation to their surrounding context.

In smart home human activity recognition, the temporal lifespan and deployment stability of recognition systems have become important research issues. Hiremath and Plötz emphasized that HAR systems in smart homes should be considered over their operational lifespan rather than only as static classifiers evaluated on fixed benchmark datasets [34]. This perspective is relevant to event-driven monitoring because real deployments require systems to handle changes in routines, environments, sensor reliability, and activity distributions over time. Similarly, Srivatsa and Plötz proposed a graph-based approach for sensor-based HAR in smart homes, showing that relationships among sensor activations can improve recognition by modelling contextual co-firing patterns rather than treating sensor events independently [35]. These studies highlight the importance of considering event structure and contextual relationships in smart home sensing. Recent work has also explored how sensor triggers can be represented in a more transferable and context-aware manner. For example, layout-agnostic HAR research has used textual descriptions of sensor triggers to capture the surrounding conditions of sensor activations and improve generalization across smart homes with different layouts [36]. This direction is particularly relevant because sensor events in homes are strongly dependent on spatial arrangement, device placement, and environmental context. Instead of assuming that raw sensor activations have the same meaning across homes, context-aware representations attempt to describe what an event means within a specific environment.

Context-aware sensing is also important from the perspective of interaction and service delivery in aging-friendly smart homes. Lu et al. reviewed and designed context-aware interactive experiences for sustainable aging-friendly smart homes, emphasizing that smart home systems should collect contextual information from multiple sources, analyse user behaviours and environmental conditions, and provide adaptive services according to the user’s situation [37]. In a related review, Lu et al. further discussed how deep learning and intelligent sensing technologies can support smart home user behaviour recognition, including optical sensors, cloud-supported data processing, and fall-related monitoring scenarios [38]. These studies indicate that context-aware smart home systems should move beyond passive data collection and toward adaptive sensing, interpretation, and feedback. Event-driven sensing is also closely related to data minimization and privacy-aware monitoring. In home environments, continuous sensing may increase privacy exposure and generate large amounts of low-value data. Context-aware systems can reduce this burden by activating sensing only when relevant events occur or when the system detects a meaningful change in the environment. For example, context-aware mental health self-tracking research has explored the use of multimodal smart speakers and home IoT data to detect contextual transitions and support self-reflection in home environments [39]. Although the application domain differs from the present study, the underlying idea is similar: home sensing systems become more useful when they can identify meaningful contextual changes rather than simply accumulate continuous data. However, existing event-driven and context-aware smart home studies often emphasize activity recognition, interaction design, or service recommendation, while giving less attention to the construction of synchronized multimodal behavioural records. In many systems, sensor events are used primarily as inputs to a classifier or as triggers for a predefined service. Less attention is paid to how event-driven sessions should be stored, how asynchronous modalities should be temporally associated, and how event records can be reused for later behavioural analysis. This gap is important for healthcare-oriented home monitoring, where the value of sensing data depends not only on immediate recognition but also on longitudinal organization, interpretability, and reusability.

The present study builds on the concept of event-driven and context-aware sensing, but extends it toward multimodal behavioural record construction. The proposed framework uses human presence detection to activate motion sessions, dining-zone occupancy to trigger meal-related image acquisition, debounce and cooldown logic to stabilize session boundaries, timestamp-based synchronization to associate motion and meal events, and structured local storage to retain session-level and episode-level records. In this sense, events are not used only as real-time triggers. They also serve as organizing units for multimodal data representation. This design is particularly suited to real home monitoring. A brief entry into the camera view, a sustained movement period, a dining-zone occupancy event, and a meal-related image sequence have different behavioural meanings and different analytical value. By explicitly modelling these events and their temporal relationships, the proposed framework transforms irregular domestic observations into structured behavioural episodes. This distinguishes the present work from conventional smart home HAR systems that focus mainly on classification accuracy and from context-aware interaction systems that focus mainly on service response. The contribution of this study lies in integrating event-driven sensing, context-aware triggering, cross-modal synchronization, and structured data representation as a deployable framework for home monitoring.

### 2.4. Dietary Context and Behavioural Monitoring

Dietary monitoring is an important component of health-related behavioural assessment because eating is closely linked to daily routines, chronic disease management, physical function, and lifestyle regulation. Traditional dietary assessment methods, such as food diaries, 24-h recalls, and food frequency questionnaires, can provide useful information but are often affected by recall bias, reporting burden, and difficulty in estimating portion size. For these reasons, image-based dietary assessment has attracted increasing attention as a less burdensome approach for recording meal-related information in daily life [40,41].

Recent studies have shown that artificial intelligence and computer vision can support several components of dietary assessment, including food recognition, food segmentation, portion-size estimation, calorie estimation, and nutrient analysis [41,42]. Systematic reviews indicate that AI-based image dietary assessment methods have the potential to reduce manual reporting burden, but they also face persistent challenges, including food occlusion, mixed dishes, lighting variation, cultural food diversity, portion-size ambiguity, and the lack of standardized validation across real-world conditions [40,41]. These challenges are particularly important for home monitoring, where meal images are captured under naturalistic conditions rather than in controlled imaging setups. In addition to conventional computer vision approaches, recent research has begun to explore multimodal large language models and vision-language models for food and nutrition analysis. For example, DietAI24 combines multimodal large language models with retrieval-augmented generation to estimate nutrients from food images while grounding the output in authoritative nutrition databases [43]. Other studies have evaluated large language models for estimating nutritional content from food photographs or have proposed reasoning-driven approaches for food energy estimation [44,45]. These studies suggest that large multimodal models may provide flexible semantic interpretation of food images, but they also show that nutritional estimation remains uncertain and requires careful validation.

For the present study, the most relevant point is not precise nutritional quantification, but the use of dietary events as behavioural context. Food images and meal-related events can provide temporal and semantic anchors for interpreting surrounding daily activity. Chung et al. showed that nutrition experts use photo-based food diaries not only to identify food items or count calories, but also to understand eating patterns over time, diet variety, eating contexts, and portion-related cues [46]. This finding is important because it suggests that meal-related images contain contextual information beyond nutrient values. In home monitoring, such context may help interpret motion data around dining-related routines, including approaching the dining table, sitting down, reaching, standing up, and post-meal movement. Mobile and app-based dietary assessment studies also show the importance of feasibility, usability, and user burden. Oei et al. evaluated an image-recognition dietary assessment app for adolescents with obesity and reported that the system was usable and acceptable, while also noting technical challenges and limitations in sustained engagement [47]. These findings suggest that dietary monitoring systems should not rely only on algorithmic estimation accuracy. They also need to consider how meal-related data are captured, how much burden is imposed on users, and how the resulting records can be used by healthcare providers or researchers.

In smart home and healthcare monitoring, dietary context can be especially valuable when combined with motion sensing. Meal-related routines are recurring daily events and often involve multiple functional behaviours, such as preparing food, carrying dishes, sitting, standing, reaching, and moving between the kitchen and dining areas. Therefore, meal events can serve as meaningful anchors for organizing motion observations. However, most image-based dietary assessment studies focus on food recognition or nutrient estimation, while most motion-monitoring studies focus on posture, gait, or activity recognition. The relationship between meal-related events and movement behaviour is less frequently treated as a unified multimodal monitoring problem. The present study addresses this gap by using dining-zone-triggered meal image acquisition as part of an event-driven multimodal sensing framework. Rather than treating dietary images as isolated inputs for precise nutrition estimation, the proposed framework uses meal events as contextual anchors for cross-modal synchronization. Representative meal images are interpreted as coarse dietary-context descriptors and linked with overlapping motion sessions through timestamp-based association. This design allows the system to construct meal-anchored behavioural episodes that combine dietary-event metadata, meal images, pose trajectories, and session-level motion records. This approach differs from conventional dietary assessment systems in two ways. First, the dietary module is not positioned as a standalone nutritional assessment tool. Instead, it provides contextual information for interpreting daily movement around meals. Second, meal events are used as temporal anchors for multimodal data organization. This is important for home monitoring because behavioural meaning often depends on context. A motion session occurring during a meal-related event may have a different interpretation from a similar motion pattern occurring outside the dining context. Therefore, integrating dietary context with motion monitoring can support richer and more interpretable behavioural records.

### 2.5. Summary of Research Gaps

Table 1 summarizes representative related studies and clarifies the positioning of the present work. Existing studies have contributed to IoT-based assisted living, smart home activity recognition, and image-based dietary estimation. However, these research directions are often developed separately. In contrast, the present study integrates event-driven motion sensing, dining-zone-triggered meal image acquisition, cross-modal temporal synchronization, structured local storage, and coarse dietary-context interpretation into a unified home-monitoring framework.

As shown in Table 1, the novelty of the present study lies in the system-level integration of sensing, event control, synchronization, and data representation. The proposed framework does not aim to replace existing activity recognition or dietary assessment methods. Instead, it provides an orchestration layer that enables heterogeneous home-monitoring observations to be activated, segmented, synchronized, and stored as interpretable behavioural episodes. This positioning directly motivates the methodology described in Section 3.

## 3. Methodology

### 3.1. Overview of the Proposed Framework

**Figure 1:**
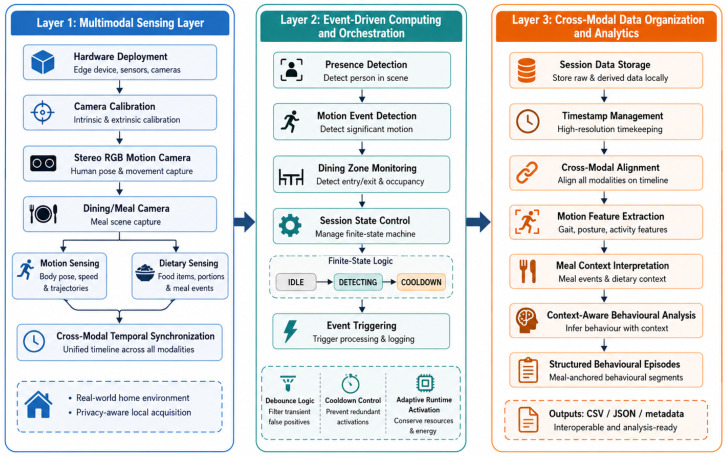
Overview of the proposed event-driven multimodal sensing and computing framework. The framework consists of three coordinated layers: multimodal sensing, event-driven computing and orchestration, and cross-modal data organization and analytics.

The proposed framework is designed to support context-aware multimodal monitoring in real-world home environments, where human behaviour is naturally irregular, temporally fragmented, and distributed across multiple daily-life events. Instead of treating home monitoring as a continuous passive recording task, the framework formulates it as an event-driven sensing and computing problem. In this formulation, sensor activation, session generation, multimodal synchronization, and structured data storage are coordinated according to detected behavioural events rather than fixed recording schedules. Figure 1 illustrates the overall structure of the proposed framework. The framework consists of three coordinated layers: a multimodal sensing layer, an event-driven computing and orchestration layer, and a cross-modal data organization and analytics layer. The multimodal sensing layer integrates stereo vision-based motion sensing and meal-related image sensing. The stereo camera is used to acquire synchronized visual streams for human presence detection, pose estimation, and three-dimensional motion reconstruction, while the dietary camera captures meal-related images when dining-related events are detected. This sensing configuration enables the system to observe both movement-related and context-related behavioural signals within the same domestic environment.

The event-driven computing and orchestration layer controls when and how the system records behavioural data. A lightweight presence-detection process first monitors whether a person is present within the target area. When valid presence or activity is detected, the system starts or maintains a motion monitoring session. When the detected target temporarily disappears, the system does not immediately terminate the session; instead, it applies debounce and cooldown logic to reduce fragmented logging caused by short occlusions, brief departures, or unstable detections. In parallel, dining-zone monitoring determines whether a person is located within a predefined meal-related region and triggers image acquisition during occupied dining periods. Through this design, the framework converts irregular domestic activity into bounded monitoring sessions that can be further analysed as behavioural episodes. The cross-modal data organization and analytics layer provides temporal indexing and structured representation of the generated observations. Motion sessions and meal-related image records are stored with timestamp information and session-level metadata. These timestamps are used to align heterogeneous observations into shared behavioural contexts. In particular, meal-related events are treated as contextual anchors rather than independent image records. Motion segments whose time windows overlap with meal-related events can therefore be grouped with dietary observations to form synchronized behavioural episodes. This organization supports later analysis of how movement patterns occur around daily routines such as meal preparation, dining, and post-meal activity.

A key design principle of the framework is separation between sensing, runtime control, and downstream analytics. The sensing layer is responsible for acquiring visual data and reconstructing motion signals. The orchestration layer manages event-triggered session control and prevents unnecessary recording. The data organization layer stores structured outputs and provides extensible interfaces for later analysis modules. This separation makes the framework modular and extensible: sensing devices, event rules, storage backends, and analytical models can be replaced or extended without redesigning the entire monitoring pipeline. The proposed framework is also designed with practical deployment constraints in mind. First, the system emphasizes local structured storage rather than continuous raw video retention, thereby reducing storage burden and limiting unnecessary exposure of privacy-sensitive domestic data. Second, it organizes monitoring data at the session and episode levels, which makes the resulting records more suitable for downstream behavioural analysis than isolated frame-level outputs. Third, it allows coarse semantic descriptors from meal-related images to be incorporated as contextual information, while leaving precise nutritional assessment and clinical interpretation to future validation studies. In this way, the framework provides a sensing and computing foundation for constructing context-rich behavioural records in real-world home environments.

### 3.2. Multimodal Sensing Layer

The multimodal sensing layer provides the physical and computational interface between the domestic environment and the event-driven monitoring framework. Its role is to acquire complementary behavioural observations from the same home context while preserving a low-burden deployment style. In the present prototype, the sensing layer consists of two visual sensing streams: a stereo RGB motion camera for human movement observation and a meal-oriented RGB camera for dietary-event image acquisition. These two streams are not intended to function as independent recording devices. Instead, they provide complementary modalities that can later be synchronized and organized into shared behavioural episodes.

#### 3.2.1. Hardware Deployment in the Home Environment

The sensing devices are deployed in a kitchen–dining environment, which represents a practical domestic space where movement, meal preparation, dining, and post-meal activity naturally occur. The body-motion camera is fixed to observe the target activity area, while the dietary camera is positioned to capture meal-related images around the dining table. This fixed-camera configuration reduces the need for wearable devices, manual sensor attachment, or explicit user operation during daily activity. The design choice of using fixed visual sensors reflects two requirements of home monitoring. First, the system should capture naturally occurring behaviour without imposing a prescribed movement task or repeated user interaction. Second, the sensing setup should support repeated monitoring over multiple days with stable camera geometry. The stereo RGB camera therefore provides a consistent view for pose estimation and three-dimensional reconstruction, while the dietary camera provides high-resolution contextual observations of meal-related events.

#### 3.2.2. Camera Calibration and Spatial Consistency

Accurate stereo-based motion reconstruction requires spatial consistency between the left and right camera views. Therefore, camera calibration is performed before monitoring starts. The calibration workflow estimates the intrinsic parameters of each camera and the extrinsic relationship between the stereo views. In the implemented prototype, a ChArUco-board-based calibration procedure is used to support reliable corner detection and camera parameter estimation under practical deployment conditions. The calibration output provides the camera matrices, distortion parameters, rotation matrix, and translation vector required for stereo triangulation. These parameters are then used to reconstruct three-dimensional body keypoints from synchronized two-dimensional pose observations. By separating calibration from runtime monitoring, the system avoids repeated calibration during daily use while maintaining a stable spatial reference for subsequent motion reconstruction.

#### 3.2.3. Stereo RGB Motion Sensing

The stereo RGB motion camera is used to capture synchronized visual streams of human activity in the monitored area. The left stream is used for lightweight person detection and presence gating, while the synchronized stereo pair is used for pose estimation and three-dimensional reconstruction. This separation allows the system to combine efficient runtime triggering with richer motion representation. For each valid motion session, the system estimates two-dimensional human pose keypoints in the left and right camera views and reconstructs three-dimensional joint trajectories through stereo triangulation. The resulting motion stream is represented as structured skeletal data rather than retained as continuous raw video. This representation is suitable for downstream analysis of body posture, movement trajectory, displacement, temporal continuity, and activity-related motion patterns. The use of stereo vision is important because many daily movements are inherently three-dimensional. A single two-dimensional camera view can capture image-plane motion, but it may lose depth-related information such as forward–backward displacement, body-centre movement, and spatial changes around the dining area. By reconstructing three-dimensional pose trajectories, the proposed sensing layer provides a more informative motion representation for later behavioural analysis.

#### 3.2.4. Meal-Oriented Dietary Sensing

The dietary sensing stream captures meal-related images when dining-related events are detected. Unlike conventional dietary assessment methods that rely on manual food diaries or retrospective questionnaires, the proposed sensing layer aims to obtain time-stamped meal-context observations within the same domestic environment as the motion stream. The dietary camera is therefore used not only to record food appearance, but also to provide contextual anchors for organizing surrounding movement episodes. In the current framework, dietary images are treated as contextual observations rather than definitive nutritional measurements. Their primary methodological role is to indicate when meal-related events occur and to provide visual evidence for coarse dietary-context interpretation. After acquisition, selected representative images can be processed by a structured image interpretation module to generate semantic descriptors, such as meal contents or approximate intake-related information. These descriptors are later associated with motion sessions through timestamp-based synchronization.

#### 3.2.5. Temporal Basis for Multimodal Organization

A key function of the sensing layer is to ensure that both motion and dietary observations can be placed on a common temporal basis. Each motion session and meal-related image record is stored with timestamp information and session-level metadata. This makes it possible to relate observations from different modalities even though they are acquired with different event conditions and sampling patterns. The motion stream is generated over time during detected human activity, whereas dietary images are captured at discrete meal-related moments. Direct frame-level correspondence between these modalities is therefore neither expected nor required. Instead, the framework uses timestamps to support episode-level organization. When a meal-related event overlaps with a motion session, the corresponding observations can be grouped into a shared behavioural context. This temporal organization provides the basis for the cross-modal synchronization mechanism described in Section 3.5.

#### 3.2.6. Privacy-Aware Local Acquisition

Because the monitoring target is a private home environment, the sensing layer is designed to limit unnecessary exposure of raw domestic data. The framework emphasizes local acquisition and local structured storage. In particular, the motion stream can be converted into skeletal coordinate files and session metadata, reducing the need to retain continuous raw video during routine operation. This design does not eliminate all privacy concerns, but it provides a practical first step toward privacy-aware multimodal monitoring. The sensing layer therefore serves three methodological purposes. First, it captures complementary movement and meal-context observations from the same domestic environment. Second, it provides calibrated stereo motion data that can be transformed into three-dimensional skeletal trajectories. Third, it generates timestamped records that support later event-driven organization and cross-modal alignment. Through these functions, the multimodal sensing layer provides the foundation for the event-driven computing and orchestration layer described in Section 3.3.

### 3.3. Event-Driven Runtime Orchestration

The event-driven runtime orchestration layer controls how the monitoring system responds to behavioural changes in the home environment. In real domestic settings, human activity does not follow a fixed experimental schedule. A person may briefly enter the monitored area, leave the scene, become partially occluded, sit at the dining table, or move between meal-related and non-meal-related contexts. A monitoring system that records continuously without runtime control may produce large amounts of redundant data, whereas a system that terminates recording immediately after each missed detection may fragment meaningful behavioural episodes. To address this problem, the proposed framework introduces an event-driven orchestration mechanism that adaptively starts, maintains, and finalizes monitoring sessions according to detected presence and context events.

The orchestration layer receives inputs from the multimodal sensing layer and converts them into runtime events. These events include human presence in the scene, motion-related activity, occupancy of the dining zone, and loss or reappearance of the target. The resulting event stream is used to control session state transitions, trigger recording, activate dietary image capture, and generate structured session metadata. In this sense, runtime orchestration serves as the computational bridge between raw sensing and higher-level behavioural episode construction.

#### 3.3.1. Presence Detection and Motion Event Monitoring

Human presence detection is used as the primary trigger for motion monitoring. A lightweight person detection process continuously evaluates whether a target person is present within the monitored area. When the person is detected with sufficient confidence, the system activates motion monitoring and begins recording the corresponding session. This design allows the system to remain responsive to behaviourally relevant events while avoiding unnecessary full processing when no person is present. Motion event monitoring is then performed during the active session. The stereo camera streams are used to obtain pose sequences and reconstruct three-dimensional skeletal trajectories. Unlike a fixed-duration recording scheme, the event-driven mechanism does not assume that all activity episodes have the same length or structure. Instead, the session remains active as long as the runtime state machine determines that the activity episode is still ongoing or likely to resume within a short time window. This strategy is important for real-world monitoring, because naturally occurring domestic movement often contains short pauses, temporary disappearance from the camera view, and intermittent re-entry into the monitored space.

#### 3.3.2. Dining-Zone Monitoring

In parallel with general presence and motion monitoring, the framework includes dining-zone monitoring to detect meal-related contexts. During system setup, a dining region is defined within the camera view. The system then evaluates whether the detected person overlaps with this predefined region. When dining-zone occupancy is detected, the dietary sensing stream is activated and meal-related images are captured according to the configured acquisition rule. The dining-zone mechanism plays two methodological roles. First, it enables the system to acquire meal-context observations without requiring the user to manually take pictures or record food intake. Second, it provides temporal anchor events for later cross-modal organization. A dining event marks a behaviourally meaningful interval around which motion sessions and dietary observations can be aligned. Therefore, the dining-zone monitor is not merely an image acquisition trigger; it is a context-detection component that supports the construction of meal-anchored behavioural episodes.

#### 3.3.3. Finite-State Session Control

The motion monitoring process is controlled by a finite-state session model. The basic state transition is represented as follows:

(1) IDLE→DETECTING→COOLDOWN→IDLE.

In the *IDLE* state, the system performs lightweight monitoring and waits for a valid presence event. Once a target person is detected, the system enters the *DETECTING* state and starts a motion monitoring session. During this state, pose estimation, three-dimensional reconstruction, timestamp logging, and structured output generation are performed. If the person is no longer detected, the system does not immediately close the session. Instead, it enters the *COOLDOWN* state, in which the current session is temporarily maintained. If the person reappears during this interval, the system returns to the *DETECTING* state and continues the same session. If no valid reappearance occurs before the cooldown interval expires, the session is finalized and the system returns to the *IDLE* state. This finite-state design reduces the unnecessary fragmentation of session records. For example, in a kitchen–dining environment, a person may temporarily move behind furniture, lean out of the camera view, or leave the monitored area for a short period before returning. Immediate termination after every missing detection would split a single behavioural episode into multiple disconnected files. The cooldown-based state model preserves the continuity of such episodes while still preventing indefinite recording.

#### 3.3.4. Debounce Logic for Robust Triggering

To improve robustness under practical home conditions, debounce logic is applied to both session activation and session termination. A valid detection must satisfy a predefined confidence threshold before it is accepted as a presence event. Similarly, absence is not declared from a single missed detection. Instead, the system requires a sequence of consecutive missed detections before transitioning out of the active detecting state. This design reduces the influence of transient detection failures, short occlusions, motion blur, or momentary pose-estimation instability. Let $m_{t}$ denote the binary result of person detection at time *t*, where $m_{t}=1$ indicates that a valid person detection is observed and $m_{t}=0$ indicates that no valid detection is obtained. The system maintains a consecutive missing count $c_{t}$, defined as

(2) $$c_{t}=\left\{\begin{array}{cc} 0, & \mathrm{if}\;m_{t}=1,\\ c_{t-1}+1, & \mathrm{if}\;m_{t}=0. \end{array}\right.$$

Absence is confirmed only when

(3) $$c_{t}\geq \theta_{\mathrm{abs}},$$

where $\theta_{\mathrm{abs}}$ is the predefined absence threshold. In the implemented prototype, the person detection confidence threshold is set to 0.3, and the absence threshold is set to 15 detection checks. These values are used to balance sensitivity to actual departures and robustness against short detection gaps.

#### 3.3.5. Cooldown Control and Session Finalization

Cooldown control is used after temporary target disappearance to determine whether the current session should be maintained or finalized. Let $t_{\mathrm{lost}}$ denote the time at which absence is first confirmed. During the cooldown interval, the system keeps the session open and waits for target reappearance. If a valid detection occurs before the elapsed cooldown time exceeds the threshold $\Delta_{\mathrm{cooldown}}$, the system resumes the same session:

(4) $$t_{\mathrm{reappear}}-t_{\mathrm{lost}}\leq \Delta_{\mathrm{cooldown}}.$$

If no valid detection occurs within the cooldown interval, the session is finalized:

(5) $$t-t_{\mathrm{lost}}>\Delta_{\mathrm{cooldown}}.$$

In the current implementation, $\Delta_{\mathrm{cooldown}}$ is set to 300 s. This value was selected to preserve continuity across short interruptions while preventing the system from maintaining inactive sessions for excessively long periods. The cooldown mechanism is particularly useful for domestic monitoring, where activity often consists of intermittent movements rather than continuous laboratory-like actions.

#### 3.3.6. Event Triggering and Runtime Adaptation

The orchestration layer generates runtime triggers based on state transitions and context events. These triggers activate or suspend different parts of the monitoring pipeline. For example, a presence event starts motion sensing and session data logging; a dining-zone occupancy event activates meal-related image acquisition; a cooldown expiration event finalizes the current session and writes metadata to local storage. Through this mechanism, the framework allocates processing and storage resources according to behavioural relevance rather than fixed time intervals. This runtime adaptation supports three practical objectives. First, it reduces redundant recording during inactive periods. Second, it preserves continuity for behaviourally meaningful sessions despite short interruptions. Third, it creates explicit session boundaries and event timestamps that can be used by the cross-modal data organization layer. The output of the orchestration layer is therefore not merely a set of recorded frames, but a structured sequence of event-bounded sessions with associated temporal metadata.

### 3.4. Dining-Zone Detection and Dietary Event Acquisition

Dining-related behaviour is an important contextual component of daily-life monitoring because meal preparation, dining, and post-meal movement often occur within a bounded temporal and spatial context. In the proposed framework, dietary sensing is not treated as an independent food-recognition task. Instead, meal-related events are used as contextual anchors that support the temporal organization of motion observations. To achieve this, the system includes a dining-zone detection mechanism that identifies when a person is located in a predefined dining region and triggers meal-related image acquisition during the corresponding occupancy period.

#### 3.4.1. Dining-Zone Definition

Before monitoring begins, the dining zone is defined within the camera view as a rectangular region of interest. This region corresponds to the table or meal-related area in the monitored kitchen–dining environment. In the current prototype, the region is specified through a graphical interface by manually dragging a bounding rectangle over the live camera preview. This design allows the system to be adapted to different home layouts without requiring geometric reconstruction of the entire room. Let *Z* denote the predefined dining-zone region and let $B_{t}$ denote the detected person bounding box at time *t*. The system evaluates whether the detected person is occupying the dining zone by calculating the overlap between $B_{t}$ and *Z*. A dining-zone occupancy event is declared when the overlap ratio satisfies

(6) $$\frac{\mathrm{Area}(B_{t}\cap Z)}{\mathrm{Area}(B_{t})}\geq \theta_{\mathrm{zone}},$$

where $\theta_{\mathrm{zone}}$ is the dining-zone overlap threshold. In the implemented prototype, $\theta_{\mathrm{zone}}$ is set to 0.5. This criterion allows partial occlusion or partial body visibility while still requiring that a substantial portion of the detected person be located inside the dining area. Compared to a strict full-body containment rule, this overlap-based definition is more robust in domestic environments, where the subject may be seated, partially occluded by the table, or only partly visible from the camera viewpoint.

#### 3.4.2. Dining Event State Model

The dining-zone monitor is controlled by a state model similar in spirit to the motion session controller, but specialized for meal-related events. The dining event state transition is represented as

(7) EMPTY→OCCUPIED→COOLDOWN→EMPTY.

In the *EMPTY* state, the system monitors the dining zone without recording meal-related images. When a person is detected inside the predefined dining region, the system enters the *OCCUPIED* state and starts a dining event session. During this state, the dietary camera captures meal-related images at a fixed acquisition interval. If the person leaves the dining zone, the system enters the *COOLDOWN* state rather than immediately terminating the meal session. If the person re-enters the zone within the timeout period, the system resumes the same dining session. Otherwise, the session is finalized and stored with its corresponding metadata. This state-based design is necessary because dining behaviour is naturally intermittent. A person may briefly leave the table to obtain utensils, prepare additional food, or move between the kitchen and dining area. Immediate termination after every short absence would fragment a single meal event into multiple records. The cooldown state therefore preserves the continuity of meal-related contexts while still preventing the system from keeping inactive sessions open indefinitely.

#### 3.4.3. Dietary Image Acquisition

When the dining zone is occupied, the system activates dietary image acquisition. In the current implementation, meal-related images are captured at a regular interval during the active dining session. The image capture interval is set to 30 s, which provides repeated observations of the meal context without producing an excessive number of images. This interval-based acquisition is intended to capture changes in the meal scene over time, including the appearance of food items, partial consumption, and the end of the meal event. Each captured image is stored with an absolute timestamp and associated with the corresponding dining session. The dietary image records therefore contain both visual information and temporal information. The visual component provides a basis for meal-context interpretation, whereas the timestamp component enables later synchronization with motion sessions. This dual role is important because the purpose of dietary event acquisition in this framework is not limited to food recognition; it also supports temporal anchoring for multimodal behavioural analysis.

#### 3.4.4. Representative Image Selection

A complete dining session may contain multiple images, many of which are visually similar or have limited additional value. To reduce redundancy and support structured downstream interpretation, the system selects representative images from each dining session. In the current design, three temporal positions are considered: the beginning, middle, and end of the dining event. These images are intended to represent the initial meal state, the intermediate consumption state, and the final meal state. The representative image selection process also considers image quality. Images with strong blur, inappropriate brightness, or insufficient food-region information are less suitable for downstream interpretation and can be excluded from analysis. This quality filtering step is particularly important in home environments, where illumination conditions, occlusion by hands or utensils, and camera viewpoint may vary during natural dining behaviour. The selected images are then passed to the dietary context interpretation module described in Section 3.7. The output is stored as a structured record associated with the dining session. In the proposed framework, this output is interpreted as a coarse contextual descriptor of the meal event rather than a precise nutritional measurement.

#### 3.4.5. Dining Events as Contextual Anchors

The most important methodological role of dining-zone detection is to generate contextual anchor events. A dining event defines a temporally bounded interval during which meal-related behaviour is occurring. Motion sessions whose timestamps overlap with this interval can be associated with the same behavioural context. This enables the system to represent motion and dietary observations as components of a shared episode rather than as independent sensor logs. Let $D_{j}=\left[t_{j,\mathrm{start}}^{D},t_{j,\mathrm{end}}^{D}\right]$ denote the temporal interval of the *j*-th dining event, and let $M_{i}=\left[t_{i,\mathrm{start}}^{M},t_{i,\mathrm{end}}^{M}\right]$ denote the temporal interval of the *i*-th motion session. A motion session is considered to overlap with a dining event when

(8) $$\max\left(t_{j,\mathrm{start}}^{D},t_{i,\mathrm{start}}^{M}\right)<\min\left(t_{j,\mathrm{end}}^{D},t_{i,\mathrm{end}}^{M}\right).$$

When this condition is satisfied, the motion session and the dining event can be grouped into a synchronized behavioural episode. This episode-level representation allows movement patterns to be interpreted in relation to meal-related contexts, such as sitting down, reaching, standing up, meal preparation, or post-meal movement. In this way, dietary event acquisition extends the monitoring system from isolated motion recording toward context-aware behavioural sensing.

#### 3.4.6. Role in the Overall Framework

Dining-zone detection and dietary event acquisition provide the contextual modality required for cross-modal behavioural organization. The motion sensing stream captures body movement and posture, while the dietary sensing stream identifies meal-related temporal contexts. Their combination allows the system to organize daily activity around meaningful domestic events. This design has three advantages. First, it reduces user burden because meal-related observations are acquired automatically when dining-zone occupancy is detected. Second, it improves interpretability because motion data can be associated with a concrete daily-life context. Third, it supports extensibility because the same event-anchoring principle can be extended to other domestic contexts, such as medication-taking, exercise, kitchen work, or sleep-related routines. Thus, the dining-zone module functions as a concrete implementation of the broader event-driven multimodal monitoring concept.

### 3.5. Cross-Modal Temporal Synchronization

The proposed framework integrates motion sensing and dietary-event sensing, but these two modalities are inherently asynchronous. The motion stream is generated continuously or semi-continuously during detected human activity, whereas meal-related images are captured only when the dining zone is occupied. Therefore, a direct frame-to-frame correspondence between the two streams is neither technically appropriate nor behaviourally meaningful. Instead, the framework adopts an event-level temporal synchronization strategy that organizes heterogeneous observations around shared behavioural contexts. The main objective of cross-modal temporal synchronization is to convert separate motion sessions and dietary records into unified behavioural episodes. In this study, a behavioural episode refers to a temporally bounded unit that contains one or more motion observations, one or more contextual meal-related records, and the metadata required to interpret their temporal relationship. This representation is designed to support context-aware monitoring, in which movement is not interpreted as an isolated signal but as an activity occurring within a surrounding daily-life context.

#### 3.5.1. Timestamp-Based Common Temporal Reference

All motion and dietary records are stored with timestamp information based on a common system clock. For motion monitoring, each session is assigned a start time, and each pose frame is stored with a relative or absolute timestamp. For dietary sensing, each meal-related image is stored with an acquisition timestamp and associated dining-session metadata. This common temporal reference allows heterogeneous data streams to be compared and aligned after acquisition. Let $M_{i}$ denote the *i*-th motion session and $D_{j}$ denote the *j*-th dining event. Their temporal intervals are defined as

(9) $$M_{i}=\left[t_{i,\mathrm{start}}^{M},t_{i,\mathrm{end}}^{M}\right],$$

(10) $$D_{j}=\left[t_{j,\mathrm{start}}^{D},t_{j,\mathrm{end}}^{D}\right],$$

where $t_{i,\mathrm{start}}^{M}$ and $t_{i,\mathrm{end}}^{M}$ represent the start and end times of the motion session, and $t_{j,\mathrm{start}}^{D}$ and $t_{j,\mathrm{end}}^{D}$ represent the start and end times of the dining event. These intervals provide the basic units for event-level synchronization.

#### 3.5.2. Meal Events as Contextual Anchors

In the proposed framework, dining events are used as contextual anchors. A meal event is behaviourally meaningful because it usually defines a sequence of related actions, such as approaching the dining area, sitting down, handling food, eating, standing up, and leaving the table. These actions may not be continuously visible or may not belong to a single predefined activity class, but they form a coherent daily-life context. Using meal events as anchors has two advantages. First, it provides a concrete temporal reference for grouping surrounding motion observations. Second, it makes the resulting behavioural records more interpretable because motion patterns can be analysed in relation to meal-related activity. For example, movement before, during, and after dining may have different behavioural meanings even if the pose trajectories contain similar local motion patterns. To support this design, each dining event can be expanded by optional pre-event and post-event buffer intervals:

(11) $${\tilde{D}}_{j}=\left[t_{j,\mathrm{start}}^{D}-\delta_{\mathrm{pre}},t_{j,\mathrm{end}}^{D}+\delta_{\mathrm{post}}\right],$$

where $\delta_{\mathrm{pre}}$ and $\delta_{\mathrm{post}}$ denote the pre-event and post-event buffer durations, respectively. These buffers allow the framework to include motion immediately before and after dining, such as entering the dining area or leaving the table. When no buffer is applied, ${\tilde{D}}_{j}$ is equivalent to $D_{j}$.

#### 3.5.3. Overlap-Based Cross-Modal Association

A motion session is associated with a dining event when their temporal intervals overlap. Using the expanded dining interval ${\tilde{D}}_{j}$, the overlap duration between $M_{i}$ and ${\tilde{D}}_{j}$ is calculated as

(12) $$O_{ij}=\max\left(0,\min\left(t_{i,\mathrm{end}}^{M},{\tilde{t}}_{j,\mathrm{end}}^{D}\right)-\max\left(t_{i,\mathrm{start}}^{M},{\tilde{t}}_{j,\mathrm{start}}^{D}\right)\right),$$

where ${\tilde{t}}_{j,\mathrm{start}}^{D}$ and ${\tilde{t}}_{j,\mathrm{end}}^{D}$ represent the start and end times of the expanded dining interval. A valid cross-modal association is established when

(13) $$O_{ij}>0.$$

This overlap-based rule is simple, transparent, and suitable for real-world deployment. It does not require the two modalities to have the same sampling frequency or frame structure. Instead, it treats each modality as a time-indexed event stream and associates observations at the episode level. This is particularly important for home monitoring, where behavioural events may be irregular and sensor streams may be activated under different runtime conditions.

#### 3.5.4. Construction of Synchronized Behavioural Episodes

When one or more motion sessions overlap with a dining event, the framework constructs a synchronized behavioural episode. Each episode contains the dining-event metadata, meal-related image records, overlapping motion-session metadata, and the corresponding pose trajectory files. The episode can be represented as

(14) $$E_{j}=\left\{D_{j},I_{j},M_{j},X_{j},C_{j}\right\},$$

where $E_{j}$ denotes the behavioural episode anchored by the *j*-th dining event; $I_{j}$ denotes the set of meal-related images; $M_{j}$ denotes the set of motion sessions overlapping with the dining event; $X_{j}$ denotes the corresponding pose trajectories; and $C_{j}$ denotes contextual descriptors generated from meal-related image interpretation and session metadata. This representation allows the system to move from independent sensor files to context-aware behavioural records. Instead of storing motion data and meal images as separate logs, the framework explicitly records their temporal relationship. This makes the data more suitable for downstream analysis, such as comparing movement characteristics across meal-related contexts, extracting pre- and post-meal movement descriptors, or examining temporal patterns of daily activity.

#### 3.5.5. Synchronization Output and Metadata

For each synchronized episode, the system stores metadata describing the temporal structure of the association. Typical metadata fields include the dining-session identifier, motion-session identifiers, start and end times, overlap duration, number of associated meal images, number of pose frames, and paths to the corresponding structured data files. These metadata are stored in a machine-readable format such as JSON, while pose trajectories are stored in structured CSV files. The use of explicit metadata has two practical benefits. First, it makes the synchronization process transparent and reproducible. The relationship between motion and dietary records can be inspected without manually searching through raw files. Second, it enables later analytical modules to access synchronized episodes directly. For example, a downstream model can load all motion trajectories associated with a given meal event, or compare episodes according to overlap duration, activity continuity, or dietary-context descriptors.

#### 3.5.6. Role in Context-Aware Home Monitoring

The cross-modal temporal synchronization mechanism is central to the proposed framework because it transforms multimodal sensing from parallel data acquisition into structured behavioural organization. Without synchronization, motion sensing and dietary sensing would remain independent streams. With synchronization, the system can represent daily behaviour as temporally organized episodes that preserve both movement information and contextual information. This design is especially important for home monitoring applications. In domestic environments, the meaning of movement often depends on context. A short walking segment, a seated posture, or a standing transition may have different interpretations depending on whether it occurs before a meal, during meal preparation, during dining, or after dining. By using meal-related events as anchors, the proposed framework provides a practical mechanism for embedding motion observations within daily-life context.

### 3.6. Structured Data Representation and Privacy-Aware Local Storage

The proposed framework is designed not only to acquire multimodal data, but also to organize the generated observations into structured records suitable for later analysis. In real-world home monitoring, continuous raw video retention may impose substantial storage requirements and may increase privacy concerns because domestic scenes often contain sensitive personal and environmental information. Therefore, the framework adopts a structured local storage strategy in which raw sensing outputs are transformed into session-level and episode-level records. This design supports downstream behavioural analysis while reducing unnecessary retention of privacy-sensitive raw data.

#### 3.6.1. Session-Level Data Organization

The basic storage unit of the framework is a monitoring session. A session is generated when the event-driven runtime orchestration layer activates a motion or dining monitoring process. Each session is assigned a unique identifier based on its start timestamp, allowing all related files and metadata to be grouped under the same directory. This organization makes it possible to manage long-term monitoring data without manually searching through continuous video files or unstructured logs. For motion monitoring, each valid session is stored as a motion-session directory. The directory contains structured pose files and session metadata. The two-dimensional pose file records the detected body keypoints in the left and right image views, while the three-dimensional pose file stores reconstructed skeletal trajectories after stereo triangulation and quality control. Each row in the pose file corresponds to a timestamped pose observation and includes joint coordinates and related frame information. For dietary monitoring, each dining event is stored as a dining-session directory. This directory contains the captured meal-related images, image timestamps, session-level metadata, and, when available, structured dietary-context interpretation results. By storing motion sessions and dining sessions as timestamped units, the framework provides a consistent basis for cross-modal temporal synchronization.

#### 3.6.2. Structured Motion Representation

The motion stream is represented as skeletal coordinate data rather than as continuous raw video. In the current implementation, human pose is represented using a 17-keypoint body model. For each detected person, two-dimensional keypoints are first estimated in synchronized camera views. These two-dimensional observations are then triangulated to obtain three-dimensional joint coordinates. The resulting motion representation can be expressed as

(15) $$P_{t}=\left[p_{t,1},p_{t,2},\ldots ,p_{t,K}\right],$$

where $P_{t}$ denotes the pose observation at time *t*, *K* denotes the number of body keypoints, and $p_{t,k}=\left(x_{t,k},y_{t,k},z_{t,k}\right)$ denotes the three-dimensional coordinate of the *k*-th keypoint. This representation preserves body-motion information while omitting most scene-level appearance information contained in raw video. The structured pose representation is useful for later computation of motion descriptors such as displacement, velocity, posture variation, centre-of-body movement, temporal continuity, and session-level activity characteristics. Because the data are stored in tabular form, they can be processed by conventional statistical tools, machine learning pipelines, or visualization modules without requiring repeated video decoding.

#### 3.6.3. Dietary Event Metadata and Context Records

Dietary-event data are also stored in structured form. Each dining session includes metadata such as session identifier, start time, end time, duration, number of captured images, image timestamps, and file paths. When representative meal images are processed by the dietary context interpretation module, the generated semantic descriptors are stored in a structured JSON format. These descriptors include recognized meal components, estimated food weights, item-level and meal-level nutrient estimates, and confidence-related fields generated by the interpretation procedure. In this framework, dietary records are not regarded as definitive nutritional ground truth. Instead, they function as contextual observations associated with meal-related events. Their structured representation allows downstream analysis modules to access meal-context information together with motion-session data. This design is particularly useful for context-aware home monitoring because it enables the system to represent not only movement itself, but also the daily-life situation in which movement occurs.

#### 3.6.4. Episode-Level Data Representation

After cross-modal temporal synchronization, motion sessions and dining events can be grouped into synchronized behavioural episodes. An episode-level record links a dining event, associated meal images, overlapping motion sessions, pose trajectory files, and derived contextual descriptors. This record can be represented as a machine-readable metadata object, for example,

(16) $$R_{j}=\left\{{\mathrm{id}}_{j},D_{j},I_{j},M_{j},F_{j},C_{j}\right\},$$

where $R_{j}$ denotes the record of the *j*-th synchronized behavioural episode, ${\mathrm{id}}_{j}$ denotes the episode identifier, $D_{j}$ denotes the dining-event interval, $I_{j}$ denotes the set of associated meal images, $M_{j}$ denotes the set of overlapping motion sessions, $F_{j}$ denotes the file paths to structured pose and metadata files, and $C_{j}$ denotes contextual descriptors derived from meal-related interpretation and session metadata. This episode-level representation is important because it transforms the output of multimodal sensing from separate modality-specific files into analysis-ready behavioural units. Such records can be directly used to examine movement around meal-related contexts, compare session-level behaviour across days, or support future modelling of daily-life behavioural patterns.

#### 3.6.5. Privacy-Aware Local Storage

Privacy is a central consideration in home-based monitoring. Domestic environments may contain identifiable persons, private objects, and daily routines that should not be unnecessarily exposed. The proposed framework therefore emphasizes local storage and structured data reduction. In routine operation, the system can store pose coordinates, timestamps, session metadata, and selected contextual images instead of retaining continuous raw video streams. This strategy provides several practical advantages. First, skeletal coordinate data contain movement-related information while reducing the amount of visual appearance information stored from the home environment. Second, local storage avoids unnecessary transmission of sensitive domestic data to external servers during data acquisition. This local-processing claim applies to real-time sensing, event control, pose reconstruction, and structured storage. For the preliminary dietary evaluation reported in this study, a limited set of representative meal images was subsequently submitted manually to a custom GPT through the ChatGPT web interface using GPT-5.3 Instant for post hoc analysis; therefore, the dietary interpretation stage was cloud-hosted rather than locally executed. web interface for post hoc analysis; therefore, the dietary interpretation stage was cloud-hosted rather than locally executed. No programmatic API or API key was used. Only the selected meal images required for dietary evaluation were submitted, whereas continuous raw video, pose trajectories, and motion-session records remained on the local device. Third, structured metadata makes it possible to inspect, filter, or delete specific sessions without searching through large raw video archives. It should be noted that structured storage does not eliminate all privacy risks. Pose trajectories, timestamps, and meal-related images may still contain sensitive information. Therefore, the proposed design should be understood as a privacy-aware data minimization strategy rather than a complete privacy-preserving solution. Future deployments should further incorporate access control, encryption, anonymization, user consent management, and clear data retention policies.

#### 3.6.6. Extensibility of the Data Layer

The data representation layer is designed to be backend-independent. Although the current prototype stores data in a local file hierarchy, the same session and episode structures can be connected to other storage backends, including relational databases, cloud storage, or secure institutional servers. The framework separates data acquisition and runtime control from downstream data management, allowing storage modules to be replaced without modifying the core sensing and orchestration logic. This extensibility is important for future intelligent monitoring applications. As the framework is extended to additional sensors or behavioural contexts, the same storage principle can be applied: each modality produces timestamped session records, and cross-modal synchronization constructs higher-level behavioural episodes. For example, future versions may incorporate wearable sensors, depth cameras, environmental sensors, medication-event logs, or self-reported context labels. These additional modalities can be integrated through the same timestamped metadata structure.

### 3.7. LLM-Assisted Dietary Context Interpretation

The final component of the methodology is an LLM-assisted module for dietary context interpretation. In the proposed framework, this module is not intended to replace clinical nutritional assessment or precise dietary measurement. Instead, its role is to convert selected meal-related images into structured semantic descriptors that can be associated with dining events and synchronized behavioural episodes. This design is consistent with the overall purpose of the framework: to support context-aware multimodal monitoring by organizing movement and meal-related observations into interpretable daily-life records.

The dietary interpreter was implemented as a custom GPT named “Meal Image Nutrition Analyzer” and was accessed manually through the ChatGPT web interface after data collection. The module was cloud-hosted rather than locally deployed, and no programmatic API or API key was used. No domain-specific fine-tuning, LoRA adaptation, or model-parameter update was performed. The dietary-analysis task was specified through a fixed zero-shot structured prompt that defined food identification, portion estimation, nutrient estimation, uncertainty reporting, and a mandatory JSON output structure. The exact backend model identifier was not logged during the original evaluation and therefore could not be retrospectively verified.

#### 3.7.1. Role of LLM-Assisted Interpretation

Meal images acquired in a home environment may contain useful dietary information, including visible food items, approximate portion sizes, and nutrient-related characteristics. Raw images alone are not directly suitable for structured behavioural analysis. They need to be transformed into machine-readable descriptors that can be stored, compared, and linked with motion data. The LLM-assisted module addresses this need by generating structured textual and numerical outputs from representative meal images.

The module is used as a semantic interpretation component rather than as a standalone food-recognition model. Its output is treated as contextual information associated with a meal event. For each meal, the module identifies visible food items, estimates item-level food weights, estimates energy and nutrient values for each food, aggregates the estimates at the meal level, and attaches prompt-defined confidence scores to the estimated fields. These numerical outputs are not treated as validated intake measurements; instead, they are retained as coarse dietary-context descriptors and linked with the corresponding dining event and synchronized motion sessions. This linkage follows the cross-modal temporal synchronization mechanism described in Section 3.5.

#### 3.7.2. Representative Image Input

Because a dining session may contain multiple captured images, directly submitting all images to an interpretation model would increase computational cost and may introduce redundant outputs. Therefore, the framework first selects representative images from each dining session. In the current design, three images are selected to represent the temporal structure of the meal event: an initial image, an intermediate image, and a final image. Let $I_{j}=\left\{I_{j,1},I_{j,2},\ldots ,I_{j,n}\right\}$ denote the set of images captured during the *j*-th dining session. The representative image set is defined as

(17) $$I_{j}^{\mathrm{rep}}=\left\{I_{j}^{\mathrm{start}},I_{j}^{\mathrm{middle}},I_{j}^{\mathrm{end}}\right\},$$

where $I_{j}^{\mathrm{start}}$, $I_{j}^{\mathrm{middle}}$, and $I_{j}^{\mathrm{end}}$ correspond to the beginning, middle, and end of the dining event, respectively. The start image captures the initial meal state, the middle image captures the intermediate state of the dining process, and the end image captures the final meal state after consumption or meal termination. To improve input quality, images with severe blur, inappropriate brightness, or insufficient food-region information can be excluded before interpretation. This pre-filtering step is useful in real domestic environments, where illumination, occlusion, tableware, hand movement, and viewpoint variation may affect image quality.

#### 3.7.3. Structured Prompting Strategy

The selected representative images were processed using a fixed zero-shot structured prompt configured in the custom GPT. The prompt required the model to integrate evidence from one to three images of the same meal, avoid duplicate counting, identify visible foods using standardized English names, and return an explicit unknown category when a food item could not be identified reliably. When portion size could not be estimated reliably, the prompt instructed the model to return a null weight value rather than fabricate an estimate. For each identified food item, the required outputs were estimated weight, energy, protein, fat, carbohydrate, sugar, and fibre, together with field-level confidence scores. Meal-level nutrient values were required to equal the sum of the corresponding item-level estimates. The prompt-defined confidence score combined visual-recognition confidence, portion-estimation confidence, and nutrition-estimation confidence using fixed weights of 40 percent, 30 percent, and 30 percent, respectively. The output was required to conform to a predefined JSON output structure containing the meal name, food-level estimates, meal-level nutrient totals, and an explicit description of the confidence rule. A simplified representation of the required JSON output structure is shown below.

{

"meal_name": "",

"foods": [

{

"food_name": "",

"confidence": 0,

"weight_g": {

"value": 0,

"confidence": 0

},

"nutrition": {

"energy_kcal": {

"value": 0,

"confidence": 0

},

"protein_g": {

"value": 0,

"confidence": 0

},

"fat_g": {

"value": 0,

"confidence": 0

},

"carbohydrate_g": {

"value": 0,

"confidence": 0

},

"sugar_g": {

"value": 0,

"confidence": 0

},

"fiber_g": {

"value": 0,

"confidence": 0

}

}

}

],

"meal_nutrition": {

"energy_kcal": {

"value": 0,

"confidence": 0

},

"protein_g": {

"value": 0,

"confidence": 0

},

"fat_g": {

"value": 0,

"confidence": 0

},

"carbohydrate_g": {

"value": 0,

"confidence": 0

},

"sugar_g": {

"value": 0,

"confidence": 0

},

"fiber_g": {

"value": 0,

"confidence": 0

}

},

"confidence_rules": {

"formula":

"0.4∗visual + 0.3∗portion + 0.3∗nutrition"

}

}

The JSON output structure shown above is an abbreviated representation of the full output format used during the evaluation; the numerical zeros are illustrative placeholders showing field locations and are not used as default fallback values for corrupted or unavailable outputs. All records used in the evaluation were manually checked for valid JSON syntax, required fields, valid data types, non-negative numerical values when present, consistency between food-level and meal-level totals where applicable, and correspondence between identified foods and visible image evidence. Only records that passed these checks were accepted for downstream storage, episode-level linkage, and quantitative evaluation. Any output failing these checks would be classified as invalid and excluded from downstream linkage and quantitative evaluation rather than being partially parsed or numerically imputed.

#### 3.7.4. Dietary Context Descriptor

For each dining event, the LLM-assisted module generates a dietary context descriptor. This descriptor is not used as ground-truth nutritional intake. Rather, it provides a coarse semantic summary of the meal-related event. The descriptor can be represented as

(18) $$c_{j}^{D}=f_{\mathrm{LLM}}\left(I_{j}^{\mathrm{rep}},q\right),$$

where $c_{j}^{D}$ denotes the dietary context descriptor of the *j*-th dining event, $f_{\mathrm{LLM}}\left(\cdot \right)$ denotes the LLM-assisted interpretation function, $I_{j}^{\mathrm{rep}}$ denotes the representative image set, and q denotes the structured prompt. The resulting descriptor is linked to the corresponding dining session and can be included in the synchronized behavioural episode record. The resulting descriptor contains identified food items, item-level weight and nutrient estimates, meal-level aggregated nutrient values, and prompt-defined confidence scores. The descriptor is linked to the corresponding dining session and included in the synchronized behavioural episode record as a coarse contextual annotation rather than as nutritional ground truth.

#### 3.7.5. Integration with Synchronized Behavioural Episodes

After the dietary context descriptor is generated, it is integrated into the synchronized behavioural episode. As defined in Section 3.5, a behavioural episode may contain a dining event, representative meal images, associated motion sessions, pose trajectories, and contextual descriptors. The LLM-assisted dietary descriptor becomes one component of this episode-level representation. This integration allows motion observations to be interpreted together with the food-item, portion, nutrient, and confidence information derived from the corresponding meal images. For example, motion occurring during a dining episode can be associated with the structured meal descriptor, while motion before or after dining can be analysed as part of a broader meal-related behavioural routine. In this way, the LLM-assisted module contributes to context-aware behavioural monitoring, not by producing a definitive nutritional diagnosis, but by adding structured dietary context to the episode-level representation.

#### 3.7.6. Limitations of LLM-Based Dietary Interpretation

Although LLM-assisted interpretation provides a flexible way to generate structured dietary context descriptors, several limitations must be acknowledged. First, meal images captured in real homes may contain occlusions, mixed dishes, reflective tableware, or incomplete views of food items. These factors can reduce the reliability of visual interpretation. Second, LLM outputs may vary depending on prompt design, image quality, and visual ambiguity. Third, estimated consumption or nutritional information should not be interpreted as clinically accurate without external validation. Therefore, the proposed framework uses LLM-assisted output conservatively. The output is treated as coarse contextual information rather than strict quantitative nutritional assessment. This interpretation is appropriate for the present system-level study because the primary objective is to demonstrate how meal-related context can be represented and linked with motion observations. Future work should improve reliability through constrained prompting, structured output validation, uncertainty estimation, top-candidate comparison, and calibration against reference dietary records.

For future automated operation, each model response should first pass structural validation against the predefined JSON output format before it is ingested into the episode-level record. A malformed response should trigger one constrained re-generation request that restates the required JSON output structure without changing the original image input. If the repeated response remains invalid, or if severe image occlusion prevents reliable interpretation, the system should retain the meal-session identifier, timestamps, and source-image paths while marking the dietary descriptor as unavailable. No nutrient values should be fabricated or replaced with default zeros in this failure state. This fallback allows dining-event storage, motion-session generation, and cross-modal synchronization to continue even when dietary interpretation fails, thereby providing graceful degradation of the dietary module rather than failure of the complete monitoring pipeline. Future implementations should also log the validation-error type, retry count, and final processing status to support fault diagnosis and reproducibility.

## 4. Experimental Evaluation

This section evaluates the proposed event-driven multimodal sensing and computing framework in a real-world home monitoring scenario. The evaluation is designed as a system-level feasibility evaluation rather than a clinical validation study or a benchmark of a single sensing algorithm. The main purpose is to examine whether the proposed framework can operate under naturalistic domestic conditions and generate structured, usable, and temporally aligned multimodal behavioural records.

To address this purpose, the evaluation uses quantitative indicators for four functional aspects of the framework: sensing-output usability, event-driven session generation, cross-modal synchronization, and local runtime/storage feasibility. First, we examine whether the event-driven runtime mechanism can convert irregular daily activity into bounded monitoring sessions. Second, we evaluate whether the generated motion streams contain sufficient valid pose data for downstream behavioural analysis. Third, we assess whether meal-related events and motion sessions can be temporally aligned into synchronized behavioural episodes. Fourth, we report storage and runtime characteristics to examine the practical feasibility of the prototype for longer-term home monitoring.

Accordingly, the evaluation metrics include session-level statistics, valid pose-frame distribution, motion–meal overlap coverage, storage overhead, and runtime feasibility indicators. These metrics do not constitute comprehensive clinical or multi-site validation, but they provide quantitative evidence for the feasibility of the proposed sensing and computing pipeline.

### 4.1. Deployment Environment and Hardware Setup

#### 4.1.1. Deployment Environment

**Figure 2:**
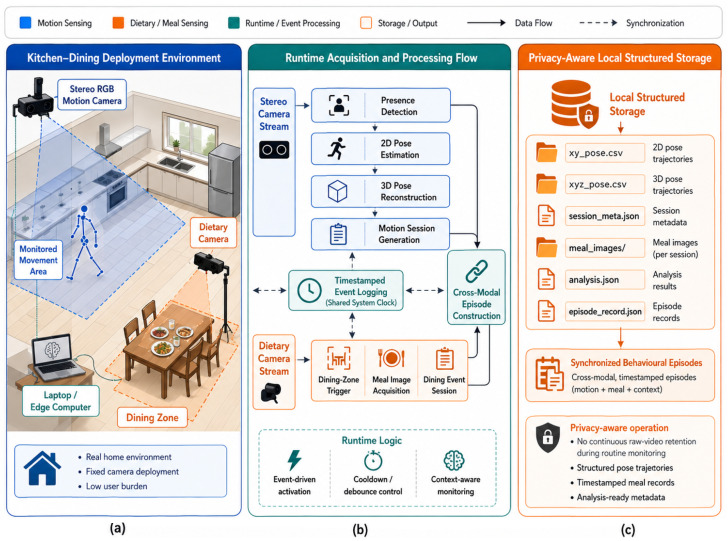
Deployment environment and hardware setup of the proposed home monitoring prototype. (**a**) Kitchen–dining deployment environment with a fixed stereo RGB motion camera, dietary camera, monitored movement area, dining zone, and local edge computer. (**b**) Runtime acquisition and processing flow for stereo motion sensing, dietary event acquisition, timestamped event logging, and cross-modal episode construction. (**c**) Privacy-aware local structured storage of pose trajectories, meal images, session metadata, and synchronized behavioural episode records.

The prototype system was deployed in a real kitchen–dining environment. This environment was selected because it represents a typical domestic space in which movement, meal preparation, dining, and post-meal activity occur naturally within a shared physical context. Unlike laboratory-based motion analysis settings, the deployment environment was not arranged as a controlled walking course or a predefined experimental task space. Instead, the system was installed to observe naturally occurring daily activity around the kitchen and dining area. The monitoring area included a movement observation region and a dining region. The movement observation region was covered by a fixed stereo RGB camera, which was used for human presence detection, two-dimensional pose estimation, and three-dimensional pose reconstruction. The dining region was monitored by a meal-oriented RGB camera, which captured meal-related images when dining-zone occupancy was detected. The two sensing streams were operated within the same domestic environment and synchronized using timestamp information. The deployment was conducted over 11 days from 3 February to 14 February 2026. During the initial phase from 3 February to 10 February, the system was used for calibration, debugging, and workflow stabilization. In this phase, simultaneous video streams were recorded to support algorithm verification, camera adjustment, and refinement of the runtime pipeline. After the system workflow was stabilized, the prototype entered real-time monitoring mode from 11 February to 14 February. The evaluation reported in this section focuses on this real-time monitoring period because it reflects the intended deployment mode of the proposed framework. During the real-time monitoring phase, the system performed human detection, pose estimation, stereo three-dimensional reconstruction, event-driven session control, and structured local data storage. To reduce storage burden and privacy exposure, continuous raw video streams were not retained during routine operation. Instead, the system stored reconstructed pose data, timestamps, session metadata, dining-event records, and selected meal-related images. Figure 2 illustrates the conceptual deployment environment and runtime data flow of the proposed prototype. The system consists of a fixed stereo RGB motion camera for movement monitoring, a meal-oriented dietary camera for dining-event acquisition, and a local laptop computer for edge-side processing and structured storage. The figure also summarizes how stereo motion sensing and dietary-event sensing are processed through timestamped event logging and organized into cross-modal behavioural episodes.

#### 4.1.2. Hardware Setup

Table 2 summarizes the hardware configuration used in the deployment. The system was operated on a general-purpose laptop computer without relying on a dedicated high-performance graphics processing unit. This configuration was selected to examine whether the proposed framework could be implemented with accessible computing resources suitable for practical home or small-scale care environments. The body-motion sensing component used a dual-lens stereo RGB camera (3D USB Camera, Shenzhen, China). The left camera stream was used for lightweight person detection and presence gating, while the synchronized stereo pair was used for pose estimation and three-dimensional reconstruction. This configuration allowed the system to combine efficient event triggering with richer spatial motion representation. The dietary sensing component used an RGB camera positioned toward the dining area to acquire meal-related images. These images were used to generate dietary-context records and to provide temporal anchors for cross-modal behavioural organization.

The hardware and camera specifications of the experimental setup are summarized in Table 2.

#### 4.1.3. Software and Runtime Configuration

The prototype was implemented in Python 3.10 under a 64-bit Windows 11 environment. Camera acquisition was implemented using OpenCV-contrib 4.8.0 with the Windows DirectShow backend, while neural-network inference was executed using ONNX Runtime 1.16.0 with the CPUExecutionProvider on the host processor.

The stereo camera delivered a composite frame of 3200 × 1200 pixels at a configured input rate of 60 fps. Each acquired frame was horizontally divided into synchronized left and right images of 1600 × 1200 pixels. Because both views originated from the same composite camera frame, they shared the same acquisition timestamp and did not require independent left–right frame matching or separate acquisition queues. Windows DirectShow provided exclusive access to the camera through a single OpenCV VideoCapture instance.

Human-presence gating was performed using a YOLOv8n detection model provided by Ultralytics (https://github.com/ultralytics/ultralytics, accessed on 10 March 2026), which was exported to ONNX format. The detector operated on sub-sampled left-view frames resized to an inference size of 192 pixels and returned person bounding boxes. A confidence threshold of 0.3 was used to accept a person detection. When presence was confirmed, two-dimensional pose estimation was performed independently for the synchronized left and right views using a YOLOv8-Pose nano model in ONNX format. The pose model used an inference size of 480 pixels and returned the 17 body keypoints defined by the COCO skeletal format. Dual-view pose inference was executed in parallel, after which corresponding two-dimensional keypoints were reconstructed in three dimensions using calibrated stereo triangulation implemented with caliscope 0.6.9.

No dedicated multi-object tracking, person re-identification, optical-flow tracking, or identity-aware temporal tracking model was used in the evaluated prototype. Under the single-user assumption, frame-level person detections and pose observations were associated with one global active session. Temporal continuity was managed at the session level through the consecutive-miss threshold, debounce logic, and the 300 s cooldown state. Therefore, references to tracking quality in the present evaluation describe the temporal continuity and usability of pose observations rather than the performance of an explicit identity-tracking algorithm.

The runtime system used a multithreaded design to prevent computational operations from blocking the graphical interface. Pose processing, three-dimensional reconstruction, and dietary-camera acquisition were executed in background worker threads, while the Tkinter main thread was reserved for interface rendering and user interaction. Communication from worker threads to the interface used a thread-safe queue.Queue. Processing modules placed status messages and results in this queue, and the Tkinter event loop polled it every 50 ms using the after() mechanism. The queue was used for asynchronous interface communication rather than for persistent buffering of the complete stereo video stream.

The configured camera input rate was 60 fps; however, the effective pose-output rate depended on CPU inference throughput and event-driven activation. During active monitoring, the measured average CPU utilization was approximately 77%, and the average memory footprint was approximately 892 MB. The configured graphical-interface update interval was 50 ms, and the average delay from an accepted person detection to motion-session activation was approximately 150 ms. Per-stage inference latency, stereo-triangulation latency, and effective end-to-end pose-processing FPS were not separately instrumented during the original deployment and are therefore not reported as measured benchmarks.

For dietary-event acquisition, the dining zone was defined as a region of interest in the camera view. When the detected person overlapped sufficiently with this region, the system started a dining-event session and captured meal-related images at the configured 30 s interval. Motion sessions and dietary-event records were assigned timestamps generated from the same local system clock, enabling later event-level cross-modal synchronization.

**Figure 3:**
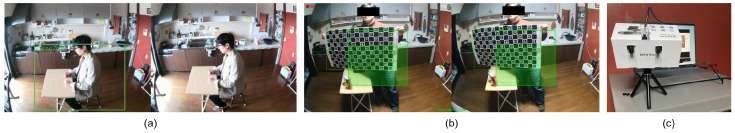
Actual prototype deployment and runtime interfaces. (**a**) Runtime monitoring interface for dining-zone definition, session triggering, and stereo camera stream inspection. (**b**) UI-guided ChArUco-based stereo calibration workflow for estimating stereo camera parameters. (**c**) Fixed stereo RGB camera and laptop setup used for local home deployment. Facial regions were anonymized to protect privacy.

The actual prototype implementation is shown in Figure 3. Figure 3a presents the runtime monitoring interface used for dining-zone definition, session triggering, and stereo camera stream inspection. Figure 3b shows the UI-guided ChArUco-based stereo calibration workflow used to estimate stereo camera parameters before deployment. Figure 3c shows the fixed stereo RGB camera and laptop setup used for local home deployment.

#### 4.1.4. Data Retention and Privacy-Aware Operation

Because the deployment environment was a real domestic space, the evaluation was conducted with attention to privacy-aware data handling. The system was designed to retain structured outputs instead of continuous raw video during the real-time monitoring phase. Motion data were stored as two-dimensional and three-dimensional skeletal coordinate files together with session metadata. Dietary records were stored as selected meal-related images and structured session information. This data retention strategy reduces the amount of identifiable visual information stored from the home environment while preserving the information required for system evaluation and downstream behavioural analysis. The retained data were organized in local directories according to session identifiers and timestamps. This structure made it possible to inspect motion sessions, dining events, and synchronized behavioural episodes without relying on continuous video archives.

### 4.2. Prototype Runtime Parameters

To improve reproducibility and clarify the operational configuration of the prototype, the main runtime parameters used in the real-time monitoring phase are summarized in Table 3. These parameters controlled event triggering, session maintenance, dining-event acquisition, and offline pose-data usability analysis. They were selected to balance responsiveness to meaningful domestic activity, robustness against transient detection failures, and the reduction in redundant sensing outputs.

### 4.3. Evaluation Metrics

Using the runtime configuration summarized in Table 3, the evaluation metrics were designed to assess the proposed framework as an event-driven multimodal sensing and computing system rather than as a benchmark of a single pose estimation model. Therefore, the metrics focus on four aspects: event-driven monitoring effectiveness, motion data usability, cross-modal synchronization utility, and practical runtime feasibility. In particular, sensing-output usability was assessed using valid-frame and valid-segment indicators, event detection was assessed using session-level and presence-interval statistics, and synchronization was assessed using motion–meal overlap measures. Runtime feasibility was assessed using local hardware requirements, structured storage cost, and the absence of continuous raw-video retention during routine monitoring. Table 4 summarizes the main evaluation dimensions and corresponding metrics used in this study.

The present evaluation did not aim to exhaustively validate each sensing component against a clinical-grade reference system. Instead, the quantitative metrics were selected to provide feasibility evidence for the main system functions. Pose-frame validity and skeletal stability were used to examine motion-sensing usability; session counts, presence intervals, and session categories were used to examine event-driven monitoring behaviour; meal-event overlap was used to examine synchronization utility; and storage rate together with hardware configuration was used to examine local deployment feasibility.

#### 4.3.1. Presence Interval Detection

Because the proposed framework uses event-driven recording with cooldown control, the raw duration of a recorded session does not necessarily correspond to continuous human presence. A session may remain open during short absences in order to avoid unnecessary fragmentation. Therefore, presence intervals are extracted from pose-frame timestamps to characterize periods of effective human activity within each recorded session. Let $t_{i}$ denote the timestamp of the *i*-th pose frame in a recorded motion session. A new presence interval is created when the temporal gap between two consecutive pose frames exceeds a predefined threshold τ:

(19) $$t_{i+1}-t_{i}>\tau .$$

In this study, τ is set to 3 s. This threshold is used to separate meaningful active periods from gaps caused by temporary absence, occlusion, or event-driven cooldown behaviour. Presence interval detection provides the basis for calculating Active Presence Duration and for characterizing the fragmentation of recorded sessions.

#### 4.3.2. Active Presence Duration

Active Presence Duration (APD) is defined as the cumulative duration of detected presence intervals within a recorded session. Let *N* denote the number of detected presence intervals in a session, and let $D_{k}$ denote the duration of the *k*-th presence interval. APD is calculated as

(20) $$\mathrm{APD}=\sum_{k=1}^{N}D_{k}.$$

APD provides a more interpretable estimate of effective human activity than raw session length. This is particularly important in the proposed framework because the cooldown mechanism may keep a session open after temporary target disappearance. In the evaluation, APD is used to distinguish brief transient triggers from sustained activity episodes.

#### 4.3.3. Valid Frame Ratio

Valid Frame Ratio (VFR) is used to evaluate the frame-level usability of reconstructed pose data. Let $N_{\mathrm{total}}$ denote the total number of pose frames in a session, and let $N_{\mathrm{valid}}$ denote the number of frames that satisfy the defined pose-quality constraints. VFR is calculated as

(21) $$\mathrm{VFR}=\frac{N_{\mathrm{valid}}}{N_{\mathrm{total}}}.$$

A pose frame is considered valid when it satisfies the following conditions: sufficient keypoint completeness, no abnormal displacement jump, and acceptable skeletal stability. In the present evaluation, VFR is interpreted as a system-level indicator of how much of the generated motion stream can be used for downstream behavioural analysis.

#### 4.3.4. Valid Segment Ratio

While VFR evaluates frame-level data usability, Valid Segment Ratio (VSR) evaluates continuity at the segment level. A valid segment is defined as a continuous sequence of pose frames that satisfies the pose-quality constraints for a minimum duration. In this study, a valid segment is defined as a sequence of at least 30 consecutive valid frames. Let $N_{\mathrm{valid}\_\mathrm{segments}}$ denote the number of valid segments, and let $N_{\mathrm{total}\_\mathrm{segments}}$ denote the total number of candidate segments in a session. VSR is calculated as

(22) $$\mathrm{VSR}=\frac{N_{\mathrm{valid}\_\mathrm{segments}}}{N_{\mathrm{total}\_\mathrm{segments}}}.$$

VSR is useful for examining whether the system can generate continuous and stable pose sequences under naturalistic home conditions. A low VSR does not necessarily indicate failure, because real domestic activity often contains occlusion, posture changes, partial body exit, and intermittent re-entry. Instead, VSR is interpreted together with VFR and session type to evaluate the practical usability of motion data.

#### 4.3.5. Session Classification Criteria

To interpret the practical behaviour of the event-driven monitoring mechanism, recorded motion sessions are classified into three categories: brief trigger, intermittent session, and sustained active session. This classification is applied during offline evaluation and is not used as an online control rule. The criteria are based on APD and pose data usability, as summarized in Table 5.

This classification enables the evaluation to distinguish low-information transient activations from sessions that contain analyzable motion data. It also allows the effectiveness of the event-driven runtime mechanism to be assessed not only by the number of sessions, but also by the distribution of analytically useful sessions and their contribution to the total pose-frame count.

#### 4.3.6. Cross-Modal Synchronization Metrics

To evaluate the utility of the cross-modal synchronization layer, meal-related events and motion sessions are compared using their timestamped intervals. A meal event is considered to have usable motion overlap when at least one motion session temporally overlaps with the meal-event interval. The number of synchronized behavioural episodes is then defined as the number of meal-anchored records that contain both dietary-event information and associated motion-session data. For each dining event $D_{j}$ and motion session $M_{i}$, the overlap duration $O_{ij}$ is calculated as described in Section 3.5. Based on these overlaps, the evaluation reports the number of meal sessions, the number of meal sessions with overlapping motion data, the number of synchronized behavioural episodes, the average overlap duration, and the percentage of meal sessions with usable motion overlap. These metrics indicate whether meal-related events can function as practical contextual anchors in a real home environment.

#### 4.3.7. Storage and Runtime Feasibility Metrics

Storage and runtime feasibility are evaluated to assess whether the prototype can support practical local operation. The storage cost of structured pose outputs is calculated at both session level and normalized level. Let $S_{i}$ denote the storage size of the *i*-th motion session and $T_{i}$ denote the duration of that session. The storage rate is calculated as

(23) $$R_{i}^{S}=\frac{S_{i}}{T_{i}}.$$

The evaluation reports the mean and median storage size per motion session, storage rate per minute, and storage size per 1000 pose frames. These normalized metrics are important because event-driven sessions vary substantially in duration. Runtime feasibility is assessed using CPU usage, memory footprint, user-interface update interval, and detection-to-session-start latency. These metrics provide initial evidence of whether the prototype can operate on a general-purpose local computing device without relying on continuous raw-video storage or high-end dedicated hardware.

#### 4.3.8. Dietary Context Estimation Metrics

The dietary context interpretation module is evaluated as a coarse contextual description component rather than as a clinical nutritional assessment tool. For representative meal images with reference nutrition labels, estimation error is calculated using Mean Absolute Percentage Error (MAPE). For a given nutritional item, MAPE is defined as

(24) $$\mathrm{MAPE}=\frac{1}{n}\sum_{r=1}^{n}\left|\frac{y_{r}-{\hat{y}}_{r}}{y_{r}}\right|\times 100\%,$$

where $y_{r}$ denotes the reference value, ${\hat{y}}_{r}$ denotes the estimated value, and *n* denotes the number of evaluated samples. In the present study, MAPE is used to quantify the approximate reliability of LLM-assisted dietary interpretation. The result is interpreted conservatively: a moderate estimation error indicates suitability for coarse dietary-context description, but not for precise nutritional quantification.

### 4.4. Event-Driven Motion Session Results

This subsection evaluates whether the proposed event-driven runtime mechanism can convert irregular domestic activity into bounded and analyzable motion monitoring sessions. The analysis focuses on the real-time monitoring period from 11 February to 14 February 2026, during which the prototype operated in the intended deployment mode. During this period, the system automatically generated motion sessions according to presence detection, debounce logic, and cooldown-based session control. The resulting sessions were analysed in terms of session count, presence interval structure, captured pose frames, valid-frame yield, and session-category distribution.

#### 4.4.1. Overview of Recorded Motion Sessions

Table 6 summarizes the overall motion-session records obtained during the real-time deployment phase. The system recorded 26 event-driven motion sessions and identified 85 presence intervals across these sessions. A total of 51,165 pose frames were generated, of which 25,925 frames satisfied the defined validity constraints. This corresponds to an overall valid-frame yield of 50.7%.

The presence of 85 intervals across 26 sessions indicates that human activity in the kitchen–dining environment was naturally discontinuous. A person could enter the monitored area, briefly leave the camera view, return to the dining region, or remain partially occluded during daily activity. Therefore, the recorded data confirm the need for explicit session management rather than simple continuous recording or immediate termination after every missed detection. The average of 3.27 presence intervals per session suggests that a single event-driven session often contained multiple active sub-periods, which is consistent with the fragmented structure of real domestic behaviour. At the same time, the system was able to convert this irregular activity into a finite set of structured motion records. From the perspective of runtime monitoring, this result is important because it shows that the proposed orchestration mechanism can generate session-level units that are suitable for later synchronization, storage, and behavioural analysis.

#### 4.4.2. Session Category Distribution

Using the classification criteria defined in Section 4.3.5, the 26 recorded motion sessions were categorized into brief triggers, intermittent sessions, and sustained active sessions. Table 7 summarizes the distribution of session categories and their contribution to the total number of captured pose frames.

Among the 26 recorded sessions, 11 sessions were classified as brief triggers, corresponding to 42.3% of all sessions. These sessions contributed only 877 pose frames, accounting for 1.7% of the total captured frames. This pattern indicates that the system remained sensitive to short appearances or transient activations, while these low-information events did not dominate the recorded dataset. Seven sessions were classified as intermittent sessions, corresponding to 26.9% of all sessions. These sessions produced 8605 pose frames, accounting for 16.8% of the total frame count. Intermittent sessions represent partially usable activity episodes but may include short interruptions, partial body visibility, or unstable pose tracking. Such sessions are expected in a real kitchen–dining environment, where the subject may sit, stand, reach, move behind furniture, or temporarily leave the monitored region. Eight sessions were classified as sustained active sessions, corresponding to 30.8% of all sessions. Although these sessions represented fewer than one-third of the recorded sessions, they produced 41,683 pose frames, corresponding to 81.5% of the total captured frames. This concentration of frame data in sustained sessions suggests that the event-driven runtime mechanism was effective in preserving behaviourally meaningful activity windows while limiting the relative contribution of short transient triggers.

#### 4.4.3. Frame Contribution by Session Type

**Figure 4:**
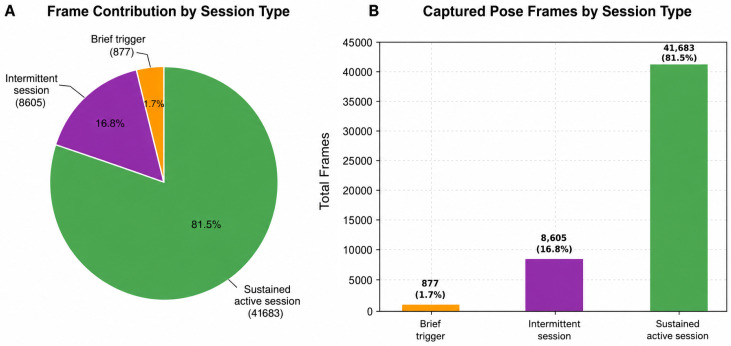
Contribution of each session category to the total number of captured pose frames. Sustained active sessions accounted for 30.8% of all sessions but contributed 81.5% of the captured pose frames, whereas brief triggers accounted for 42.3% of sessions but contributed only 1.7% of captured frames.

Figure 4 visualizes the contribution of each session category to the total number of captured pose frames. The result shows a highly uneven distribution: brief triggers were frequent but contributed little data, whereas sustained active sessions were fewer but contributed the majority of captured frames.

This distribution supports the methodological value of event-driven session control. In a naive continuous recording strategy, all periods of camera operation may be retained regardless of behavioural relevance. In contrast, the proposed framework uses runtime presence detection, debounce logic, and cooldown control to form session records that reflect the temporal structure of natural activity. The resulting session distribution indicates that most analyzable pose data were concentrated in sustained activity periods rather than dispersed across low-value transient activations. This finding is particularly important for home monitoring applications because useful behavioural information is often sparse and context-dependent. The proposed mechanism does not simply reduce recording time; it organizes the monitoring output into session categories with different analytical value. Brief triggers can be retained as evidence of system responsiveness, intermittent sessions can be inspected for partially usable activity, and sustained active sessions can serve as the main source for subsequent motion analysis.

#### 4.4.4. Implications for Event-Driven Home Monitoring

The motion-session results provide initial evidence that the proposed framework can operate under naturalistic domestic conditions and generate structured behavioural records. The 26 recorded sessions show that the system was able to detect and record daily activity without requiring the user to initiate each session manually. The 85 presence intervals further indicate that the system captured fragmented and intermittent behaviour, which is typical of real home environments. The most important result is that sustained active sessions contributed most of the captured pose data. This suggests that the runtime mechanism did not merely create arbitrary recording files, but helped organize irregular activity into records with different levels of analytical value. From a system-level perspective, this supports the feasibility of event-driven orchestration as a practical strategy for long-term home monitoring. Taken together, these results provide quantitative feasibility evidence for the event-driven monitoring mechanism. In particular, the combination of 26 motion sessions, 85 presence intervals, and the concentration of captured frames within sustained active sessions indicates that the runtime logic organized fragmented domestic presence into bounded and behaviourally meaningful monitoring records. The next subsection further examines the quality and usability of the generated pose frames, including valid-frame distribution and continuity of stable pose tracking.

### 4.5. Captured and Valid Pose Frame Distribution

This subsection further examines the distribution of captured pose frames across different motion-session categories. While the previous subsection focused on how event-driven sessions were generated and classified, the present analysis evaluates whether the generated pose data were concentrated in sessions that are meaningful for downstream behavioural analysis. In the proposed framework, a valid pose frame refers to a frame that remains usable after pose extraction and quality screening. Such frames provide the basic unit for later motion-feature computation, including body displacement, posture variation, trajectory continuity, and session-level activity descriptors.

#### 4.5.1. Overall Distribution of Captured Pose Frames

Across the 26 recorded motion sessions, the system generated 51,165 captured pose frames, of which 25,925 frames satisfied the defined validity constraints. The captured frames were not evenly distributed across session categories. As summarized in Table 8, brief triggers accounted for 42.3% of all sessions but contributed only 877 captured pose frames, corresponding to 1.7% of all captured pose frames. In contrast, sustained active sessions accounted for 30.8% of all sessions but contributed 41,683 captured pose frames, corresponding to 81.5% of all captured pose frames.

**Figure 5:**
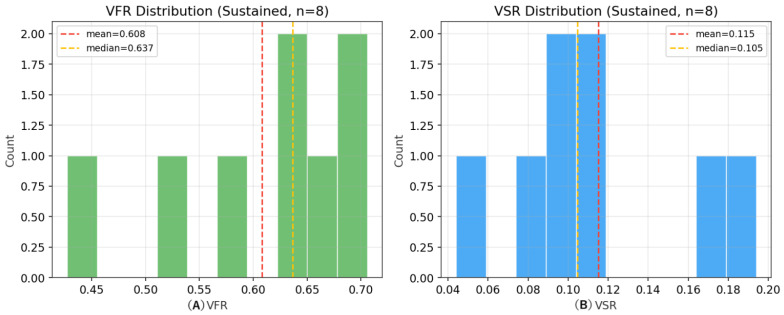
Distribution of Valid Frame Ratio (VFR) and Valid Segment Ratio (VSR) across sustained active sessions. (**A**) VFR distribution showing frame-level pose validity. (**B**) VSR distribution reflecting the continuity of stable pose tracking segments.

The distribution indicates that the majority of captured motion data were concentrated in sustained active sessions. The mean number of captured pose frames per sustained active session was 5210.4, which was substantially higher than that of intermittent sessions and brief triggers. In comparison, intermittent sessions contained an average of 1229.3 captured pose frames per session, whereas brief triggers contained only 79.7 captured pose frames per session. This result suggests that session type is strongly associated with the amount of motion information generated by the system. Figure 5 further summarizes the distribution of VFR and VSR across the eight sustained active sessions. The VFR distribution shows that sustained sessions generally retained a moderate level of frame-level pose validity, with a mean VFR of 0.608 and a median VFR of 0.637. The VSR distribution shows that continuous stable pose segments were more limited, with a mean VSR of 0.115 and a median VSR of 0.105. This result is consistent with the nature of real home environments, where occlusion, seated posture, partial body visibility, and temporary body rotation can interrupt continuous pose tracking even when frame-level pose detection remains usable.

#### 4.5.2. Skeletal Stability Across Body Regions

In addition to frame-level validity and segment-level continuity, skeletal stability was examined using the coefficient of variation (CV) of reconstructed bone lengths. Since anatomical bone lengths should remain relatively stable within a session, large CV values indicate instability in pose reconstruction, depth triangulation, partial occlusion, or inconsistent joint localization. Therefore, bone-length CV provides a practical quality indicator for evaluating three-dimensional skeletal reconstruction under naturalistic home conditions.

**Figure 6:**
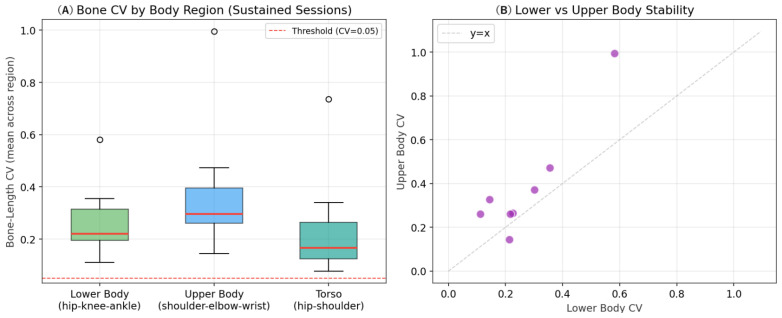
Analysis of skeletal stability across body regions during sustained active sessions. (**A**) Bone-length coefficient of variation (CV) across lower-body, upper-body, and torso-related segments. (**B**) Comparison of lower-body and upper-body CV values for individual sustained active sessions.

Figure 6 shows the distribution of bone-length CV values across lower-body, upper-body, and torso-related segments during sustained active sessions. The results indicate that skeletal stability differed across body regions. Lower-body and torso segments showed comparatively lower CV values in most sessions, whereas upper-body segments tended to show higher variability. This pattern is reasonable in a kitchen–dining environment because upper-limb movements such as reaching, eating-related gestures, arm occlusion, and partial visibility near the table can introduce instability into wrist, elbow, and shoulder keypoint estimation.

The comparison between lower-body and upper-body CV values further suggests that reconstruction quality should be interpreted at the body-region level rather than only at the whole-body level. Even within sustained active sessions, some body regions may provide more stable motion information than others. This finding supports the use of region-aware quality assessment when extracting downstream behavioural features from pose trajectories.

Although lower-body segments exhibited comparatively lower CV values than upper-body segments in most sustained active sessions, elevated lower-body variability was observed in several individual sessions. Partial visibility and furniture occlusion may contribute to lower-limb reconstruction instability, particularly during seated postures and sit-to-stand transitions. However, bone-length CV is a composite stability indicator and does not isolate occlusion as the sole source of reconstruction error; camera viewpoint, stereo triangulation uncertainty, and inconsistent two-dimensional keypoint localization may also contribute to the observed variability. These findings should therefore be interpreted as evidence of body-region-dependent reconstruction reliability rather than as direct quantitative validation of robustness to lower-body occlusion.

#### 4.5.3. Relationship Between Session Type and Data Usability

The observed frame distribution provides practical evidence that the event-driven monitoring mechanism did not merely detect human presence, but also separated low-information transient events from higher-value activity episodes. Brief triggers were relatively frequent, but their contribution to the total captured pose-frame count was minimal. These sessions likely corresponded to short appearances, temporary entries into the camera view, or non-sustained activations. Although they are useful for verifying system responsiveness, they provide limited motion continuity for downstream behavioural analysis. Intermittent sessions represented an intermediate category. They contributed 8605 captured pose frames, or 16.8% of the total captured frames. These sessions may contain useful activity segments, but their temporal structure is more fragmented than that of sustained active sessions. In real home environments, such fragmentation may be caused by partial occlusion, sitting and standing transitions, temporary departure from the monitored area, or repeated movement between the kitchen and dining regions. Sustained active sessions represented the main source of analyzable pose data. Although only 8 of the 26 sessions were classified as sustained active sessions, they contributed more than four-fifths of all captured pose frames. This concentration is important because downstream motion-feature extraction requires continuous or semi-continuous pose sequences. The result therefore supports the use of sustained active sessions as the primary analytical unit for later behavioural modelling.

#### 4.5.4. Frame Concentration Effect of Event-Driven Monitoring

Figure 4 shows that captured pose frames were highly concentrated in sustained active sessions. This concentration effect is a desirable property of the proposed event-driven framework. In a real home environment, not every person detection event corresponds to meaningful movement data. A system that treats all detections equally may generate a large number of short and low-value records. By contrast, the proposed framework uses session-level classification to identify which recordings are likely to contain sufficient motion continuity for analysis. To quantify this concentration effect, the average captured-frame count per sustained active session was approximately 65.4 times higher than that of a brief trigger session:

(25) $$\frac{5210.4}{79.7}\approx 65.4.$$

Similarly, the average captured-frame count per sustained active session was approximately 4.2 times higher than that of an intermittent session:

(26) $$\frac{5210.4}{1229.3}\approx 4.2.$$

These ratios indicate that sustained active sessions were not only longer or more frequent in terms of frame count, but also substantially more informative as units of motion data collection. Therefore, the event-driven session structure provides a useful basis for prioritizing data analysis. For example, sustained active sessions can be selected for detailed feature extraction, intermittent sessions can be inspected for partial motion patterns, and brief triggers can be retained as metadata-level evidence of transient activity.

#### 4.5.5. Implications for Downstream Motion Analysis

The captured-frame distribution and pose-quality metrics have several implications for downstream analysis. First, the concentration of captured frames in sustained active sessions, together with the VFR and VSR results, suggests that event-driven monitoring can reduce the analytical burden by identifying high-value sessions. Instead of processing all detected events with equal priority, later analysis can focus on sessions that contain sufficient pose-frame continuity. Second, the distribution supports the use of session-level and episode-level representations. Because captured pose frames are clustered in specific sessions, behavioural analysis can be performed at the session level rather than at the isolated frame level. This is consistent with the overall design of the proposed framework, which aims to convert irregular domestic activity into structured behavioural records. Third, the results indicate that pose data quality should be interpreted together with behavioural context. A low number of captured or valid frames in brief triggers does not necessarily indicate system failure. In many cases, it reflects the fact that the person only appeared briefly in the monitored area. Therefore, frame count and session category should be considered jointly when evaluating home-monitoring data.

#### 4.5.6. Summary of Pose-Frame Findings

The captured pose-frame analysis shows that the event-driven framework generated a total of 51,165 captured pose frames during the real-time monitoring period, of which 25,925 frames satisfied the defined validity constraints. The captured frames were highly concentrated in sustained active sessions, which accounted for 81.5% of all captured pose frames despite representing only 30.8% of the sessions. This finding supports the feasibility of the proposed framework for concentrating analyzable motion data within behaviourally meaningful activity windows. It also demonstrates that event-driven session organization can help distinguish transient activations from sessions that provide sufficient motion continuity for downstream behavioural analysis. From the perspective of sensing-output validation, the valid-frame and valid-segment results indicate that the prototype generated pose streams that were partially usable for downstream behavioural analysis under naturalistic home conditions. However, these metrics should be interpreted as usability indicators rather than as validation against a motion-capture ground truth system.

### 4.6. Cross-Modal Overlap Between Motion and Meal Events

This subsection evaluates whether meal-related events can function as practical contextual anchors for organizing motion observations in a real home environment. In the proposed framework, motion sessions and dining events are generated by different runtime conditions. Motion sessions are activated by human presence and movement in the monitored area, whereas dining events are activated by occupancy of the predefined dining zone and meal-oriented image acquisition. Therefore, the key question is whether these two event streams can be temporally associated to form synchronized behavioural episodes.

#### 4.6.1. Overview of Meal-Related Event Records

During the real-time monitoring period, the system obtained eight meal-related event records. Each meal event was stored with a dining-session identifier, image acquisition timestamps, and session-level metadata. These records provided the temporal basis for linking meal-context observations with motion-session data. A meal event was considered to have motion overlap when at least one motion session overlapped with the corresponding dining-event interval according to the overlap rule defined in Section 3.5. Table 9 summarizes the cross-modal overlap between meal events and motion sessions. Among the eight meal-related event records, seven had temporal overlap with at least one motion monitoring session. This corresponds to an overlap coverage of 87.5%. Only one meal event did not have an overlapping motion session.

The result indicates that most meal-related events occurred within time windows that could be associated with motion monitoring data. This is important because the proposed framework does not assume continuous recording of all modalities. Instead, the two streams are activated by different event conditions and are later associated using timestamp information. The high overlap coverage suggests that the event-driven sensing design was able to capture meal-context observations together with surrounding human movement in most recorded meal events.

#### 4.6.2. Meal Events as Practical Contextual Anchors

The observed overlap supports the use of meal-related events as contextual anchors for home monitoring. A meal event provides a meaningful daily-life reference point around which motion data can be organized. For example, motion segments occurring before, during, or after a meal event may correspond to approaching the dining table, sitting down, reaching, standing up, meal preparation, or post-meal movement. These behaviours are difficult to interpret from pose trajectories alone, but become more meaningful when they are linked to a dining context. In the proposed framework, a synchronized behavioural episode is constructed when a meal event and one or more motion sessions overlap temporally. The episode contains the dining-event metadata, representative meal images, associated motion-session identifiers, pose trajectory files, and dietary-context descriptors. This episode-level representation makes it possible to move beyond isolated sensor streams and organize daily-life observations into interpretable multimodal records. The seven overlapping meal events therefore provide evidence that the proposed framework can generate meal-anchored behavioural episodes under naturalistic domestic conditions. These episodes are not intended to provide clinical conclusions by themselves. Rather, they establish a structured data basis for future analysis of daily routines, movement around meals, and potential digital behavioural biomarkers.

#### 4.6.3. Interpretation of the Non-Overlapping Meal Event

One meal-related event did not temporally overlap with any motion monitoring session. This type of non-overlap is expected in real home environments and does not necessarily indicate system failure. Several practical factors may lead to non-overlap. First, the dietary camera may capture a meal-related scene while the person is outside the motion camera’s effective field of view. Second, the subject may remain seated or partially occluded in a position where motion detection or pose reconstruction is unstable. Third, the motion session may have been finalized before or after the dining event because of the runtime state transition and cooldown rules. This non-overlapping case highlights an important characteristic of event-driven multimodal monitoring: each modality may be activated by different contextual conditions. Therefore, complete overlap between all modalities is not always expected. Instead, the purpose of the framework is to retain timestamped event records and explicitly identify which events can be synchronized. Non-overlapping records are also informative because they indicate situations where sensor placement, dining-zone definition, or event-triggering rules may need further refinement.

#### 4.6.4. Contribution to Context-Aware Behavioural Records

The motion–meal overlap results demonstrate the feasibility of constructing context-aware behavioural records from asynchronous event streams. The 87.5% meal-event overlap coverage suggests that the dining-zone trigger frequently captured meal-related contexts that were temporally associated with motion monitoring. This supports the methodological assumption that meal events can serve as anchors for organizing movement data in the home. From a data-organization perspective, the overlap results also validate the use of timestamped metadata. Because motion sessions and dining events were stored with their own session identifiers and timestamps, their relationship could be reconstructed after acquisition without requiring direct frame-level correspondence. This is suitable for real-world home monitoring, where continuous synchronized recording of all modalities may be impractical or privacy-sensitive. From an application perspective, synchronized meal-anchored episodes may support future analyses of daily-life functional behaviour. For example, these episodes could be used to compare movement before and after meals, examine activity continuity during dining-related routines, or relate motion patterns to coarse dietary-context descriptors. Such analyses require further validation with larger datasets and health-related reference measures, but the present results show that the proposed framework can generate the required multimodal data structure.

#### 4.6.5. Summary of Cross-Modal Overlap

The cross-modal overlap analysis showed that seven of eight meal-related event records overlapped with motion monitoring sessions, corresponding to an overlap coverage of 87.5%. This result indicates that the event-driven sensing framework can associate meal-context observations with motion data in most recorded meal events. The resulting synchronized behavioural episodes provide an analysis-ready representation that combines motion trajectories, meal-event records, timestamps, and contextual descriptors. These findings support the feasibility of meal-event anchoring as a practical strategy for context-aware multimodal home monitoring. The overlap analysis therefore provides quantitative evidence for the synchronization strategy. The observation that seven of eight meal-related event records overlapped with motion monitoring sessions suggests that meal events can serve as practical temporal anchors for organizing surrounding motion observations in this deployment setting.

### 4.7. Storage Efficiency and Runtime Feasibility

This subsection evaluates the practical feasibility of the proposed framework from the perspectives of local storage efficiency and runtime operation. In real-world home monitoring, a system that continuously records raw video streams may rapidly accumulate large amounts of privacy-sensitive data and impose substantial storage and management burdens. Therefore, the proposed framework was designed to retain structured motion and event records rather than continuous raw video during routine operation. The evaluation in this subsection focuses on whether this design can support practical long-term monitoring with accessible local computing resources.

#### 4.7.1. Structured Storage Strategy

During the real-time monitoring phase, the system stored reconstructed pose trajectories, timestamps, session metadata, dining-event records, selected meal-related images, and synchronized episode metadata. Continuous raw video streams were not retained during routine monitoring. This storage strategy reflects the privacy-aware data minimization principle described in Section 3.6. For motion monitoring, the main structured outputs were two-dimensional pose files, three-dimensional pose files, and session metadata files. The two-dimensional pose files stored image-plane keypoint coordinates detected from the stereo camera views, whereas the three-dimensional pose files stored reconstructed skeletal trajectories after stereo triangulation. Session metadata included session identifiers, timestamps, duration, presence-interval information, and file paths. For dietary-event monitoring, the retained data consisted of meal-related images, dining-session metadata, and structured dietary-context descriptors when available. This organization has two practical advantages. First, it separates the information required for behavioural analysis from the raw visual appearance of the home environment. Second, it makes the output data directly accessible for downstream processing without requiring repeated video decoding. As a result, the framework produces analysis-ready records while reducing the amount of data that must be retained and inspected.

#### 4.7.2. Storage Cost of Structured Pose Outputs

The storage cost of structured pose outputs was evaluated using the size of generated pose and metadata files. In the current implementation, structured pose outputs required approximately 550 kB per minute of recorded pose data. This storage rate is substantially smaller than what would be expected from retaining continuous stereo raw video streams, especially when both left and right camera views are recorded at high resolution. Let $S_{i}$ denote the storage size of the structured pose output for the *i*-th motion session, and let $T_{i}$ denote the corresponding session duration in minutes. The normalized storage rate is calculated as

(27) $$R_{i}^{S}=\frac{S_{i}}{T_{i}},$$

where $R_{i}^{S}$ represents the storage cost per minute for the *i*-th session. In the present evaluation, the observed storage rate was approximately

(28) $$R^{S}\approx 550\;\mathrm{kB}/\min.$$

This result suggests that structured skeletal data can provide a compact representation of motion activity. For example, one hour of recorded structured pose data would require approximately

(29) 550kB/min×60min=33,000kB≈33MB.

This estimate indicates that the structured pose representation is feasible for multi-day or longer-term monitoring, particularly when combined with event-driven activation. Because the system records motion sessions only when behaviourally relevant events are detected, the total storage requirement is further reduced compared to a continuous recording strategy.

#### 4.7.3. Comparison with Continuous Raw-Video Retention

The proposed framework does not require continuous raw-video retention during routine operation. This distinction is important because raw video streams contain rich visual appearance information, including identifiable persons and private domestic scenes. Retaining continuous raw video would increase both storage cost and privacy risk. In contrast, the structured pose representation stores skeletal coordinates and timestamps. Although pose trajectories may still contain behavioural information and should be handled carefully, they contain substantially less scene-level visual appearance information than raw video. This makes structured pose storage more suitable for privacy-aware home monitoring. The storage advantage is also important for data management. Structured CSV and JSON files are easier to index, filter, inspect, and process than large video archives. For example, a downstream analysis module can load a specific motion session based on its identifier, retrieve the associated three-dimensional pose trajectory, and link it to a synchronized dining event without scanning continuous video files. This improves reproducibility and reduces the manual burden of analysing long-term monitoring data.

#### 4.7.4. Runtime Feasibility on Local Computing Hardware

The prototype was operated on an HP computer equipped with an Intel(R) Core(TM) Ultra 5 125U processor, 16.0 GB RAM, Intel Graphics, and a 477 GB SSD (Intel Corporation, Santa Clara, CA, USA). No dedicated high-performance GPU was used during the real-time deployment phase. This hardware configuration was selected to evaluate whether the system could run on accessible local computing resources rather than requiring specialized laboratory equipment. The runtime pipeline included camera input, person detection, pose estimation, stereo reconstruction, dining-zone monitoring, session-state control, timestamp logging, and local structured storage. The system operated locally, and the event-driven design reduced unnecessary processing during inactive periods. In particular, lightweight person detection and dining-zone monitoring were used to determine when full motion-session processing or meal-image acquisition should be activated. Table 10 summarizes the storage and runtime feasibility indicators observed in the deployment. The results indicate that the prototype could support local sensing, event-driven processing, and structured storage using accessible hardware.

#### 4.7.5. Role of Event-Driven Operation in Storage Reduction

The storage efficiency of the proposed framework is achieved not only by storing structured pose trajectories, but also by limiting recording to event-driven sessions. In a continuous monitoring design, the system would need to process and store data regardless of whether the target person is present or whether the activity is behaviourally meaningful. By contrast, the proposed runtime orchestration layer activates motion monitoring according to human presence and manages session continuity using debounce and cooldown logic. This event-driven design has two effects. First, inactive periods are not converted into large structured pose files. Second, recorded motion data are organized into session-level units that can be selectively analysed. As shown in Section 4.4, sustained active sessions contributed most of the captured pose frames, whereas brief triggers contributed only a small proportion. Therefore, the framework can prioritize high-value motion records while retaining low-information events as lightweight session metadata. The same principle also applies to dietary-event acquisition. Meal-related images are captured only when the dining zone is occupied, rather than continuously photographing the dining table. This reduces redundant image acquisition and links dietary records to meaningful event intervals.

#### 4.7.6. Practical Implications for Home Monitoring

The storage and runtime results support the practical feasibility of the proposed framework for home monitoring. The structured pose storage rate of approximately 550 kB/min indicates that skeletal motion records can be retained over extended periods without requiring large video archives. The ability to operate on a general-purpose laptop computer further suggests that the system can be deployed in small-scale home or care environments without specialized computing infrastructure. The privacy-aware storage strategy also improves practical deployability. By avoiding continuous raw-video retention, the system reduces unnecessary exposure of visual domestic scenes. At the same time, the retained pose trajectories, meal-event records, and metadata remain suitable for downstream analysis. This balance between data utility and data minimization is important for long-term monitoring applications involving private living spaces.

#### 4.7.7. Summary of Storage and Runtime Feasibility

The storage and runtime feasibility analysis showed that the proposed framework can generate compact structured records during real-time home monitoring. Structured pose outputs required approximately 550 kB/min, corresponding to approximately 33 MB per hour of recorded pose data. The prototype operated locally on a general-purpose laptop computer without relying on a dedicated high-performance GPU. Together with event-driven activation and the absence of continuous raw-video retention during routine monitoring, these results support the feasibility of the proposed framework as a practical sensing and computing foundation for privacy-aware context-aware home monitoring. Nevertheless, the present evaluation should be interpreted as a feasibility assessment rather than a complete runtime benchmark. Although CPU utilization, memory footprint, interface-update interval, and detection-to-session-start latency were recorded, the original deployment did not separately instrument per-stage model-inference latency, stereo-triangulation latency, effective end-to-end pose-processing FPS, power consumption, or long-term performance degradation. These indicators should be evaluated systematically in future controlled runtime benchmarks.

### 4.8. Dietary Context Estimation Results

This subsection evaluates the LLM-assisted dietary context interpretation module. The purpose of this evaluation is not to validate a nutritional assessment method, but to examine whether representative meal images can provide coarse dietary-context descriptors for synchronized behavioural episode construction. Therefore, the results are interpreted from the perspective of context-aware multimodal monitoring rather than clinical dietetics.

#### 4.8.1. Evaluation Purpose

In the proposed framework, dietary-event sensing provides contextual information associated with meal-related activity. The meal images acquired during dining events are used to describe the surrounding context of movement observations, such as whether a motion session occurred before, during, or after a meal-related event. The LLM-assisted interpretation module is therefore evaluated as a semantic context-generation component. This design is different from conventional dietary assessment methods that aim to estimate exact nutrient intake. In a real home environment, food images may contain mixed dishes, occluded portions, variable illumination, and incomplete views of tableware or food items. These factors make precise nutritional quantification difficult from images alone. For this reason, the proposed framework uses LLM-assisted outputs conservatively as coarse dietary-context descriptors.

#### 4.8.2. Structured Dietary Context Output

For each evaluated dining event, one to three representative meal images were processed together using the fixed structured prompt. The generated JSON record contained identified food items, item-level weight estimates, item-level energy and nutrient estimates, meal-level aggregated nutrition values, and prompt-defined confidence scores. These structured outputs were stored together with the corresponding dining-session metadata and could subsequently be linked with overlapping motion sessions through the cross-modal synchronization mechanism described in Section 3.5. Although the records contained numerical nutrient estimates, their role in the present framework was coarse contextual annotation rather than definitive dietary intake measurement.

#### 4.8.3. Estimation Accuracy Against Reference Labels

To obtain an initial indication of the reliability of the MLLM-assisted dietary interpretation, the estimated meal-level nutrient values were compared to reference values transcribed from the original nutrition records and manually verified before analysis. MAPE was then calculated from the verified reference values and the corresponding model-generated estimates. The metric definition used for this calculation is provided in Section 4.3.8. The LLM-assisted analysis achieved an overall MAPE of 25.44% (Table 11).

The observed MAPE indicates that LLM-assisted image interpretation can provide approximate dietary-context information, but the estimation accuracy is not sufficient for strict nutritional assessment. This result supports the methodological decision to treat dietary outputs as contextual descriptors rather than as clinical intake measurements. In other words, the module can help identify and summarize meal-related contexts, but its numerical estimates should not be interpreted as definitive dietary records.

#### 4.8.4. Interpretation as Contextual Information

The dietary estimation result should be interpreted in relation to the goal of the overall framework. The framework aims to organize domestic behaviour into structured multimodal records. For this purpose, approximate information about meal-related events can still be useful, even when precise nutrient estimation is not achieved. For example, the system can identify that a meal event occurred, associate the event with representative meal images, and provide a structured description of the visible meal context. This information can then be used to interpret surrounding motion sessions. From this perspective, a moderate MAPE does not invalidate the dietary module. Instead, it clarifies its appropriate role. The module is useful for generating coarse semantic context, such as the presence of meal-related activity or approximate changes in the dining scene, but it should not be used alone for dietary diagnosis, nutritional intervention, or clinical dietary assessment. This distinction is important for avoiding overclaiming and for maintaining consistency with the system-level scope of the present study.

#### 4.8.5. Contribution to Cross-Modal Behavioural Episodes

The dietary context estimation module contributes to the construction of synchronized behavioural episodes in three ways. First, it transforms meal images into structured descriptors that can be stored together with dining-session metadata. Second, it provides contextual labels that can be associated with overlapping motion sessions. Third, it supports later analysis of movement patterns around meal-related events. For example, a synchronized behavioural episode may contain a dining-event interval, representative meal images, a dietary-context descriptor, one or more overlapping motion sessions, and corresponding pose trajectories. This structure allows movement data to be interpreted together with meal-related context. Such episode-level organization is more informative than storing motion and meal images as independent files. The dietary context module therefore extends the framework from motion monitoring to context-aware behavioural monitoring. Although the nutritional estimates are not sufficiently precise for clinical use, the structured descriptors provide useful semantic information for organizing daily-life activity records.

#### 4.8.6. Limitations of Dietary Context Estimation

Several limitations should be noted. First, the evaluation of dietary interpretation was limited in scale and should be considered preliminary. Second, the use of representative images may overlook fine-grained changes that occur between the selected start, middle, and end images. Third, the accuracy of LLM-assisted interpretation may be affected by image quality, camera angle, lighting, occlusion, dish complexity, and ambiguity in visible food items. Fourth, reference nutrition labels may not fully capture the uncertainty of real meal consumption in a home environment. These limitations suggest that future work should validate dietary interpretation using larger meal datasets, controlled reference measurements, multiple food categories, and repeated evaluation across different home environments. Future extensions may also combine image-based interpretation with additional information, such as user confirmation, food-weight records, recipe information, or temporal consumption patterns. A further reproducibility limitation is that the exact backend model identifier used by the custom GPT was not recorded during the original dietary evaluation and could not be verified retrospectively. Future evaluations should record the model identifier, access date, prompt version, and output-validation procedure, or use a locally versioned model to improve model-level reproducibility.

#### 4.8.7. Summary of Dietary Context Estimation

The dietary context estimation results show that the LLM-assisted module achieved an overall MAPE of 25.44% against reference nutrition labels. This result indicates that the module is suitable for generating coarse dietary-context descriptors, but not for precise nutritional quantification. Within the proposed framework, this level of performance is appropriate because the dietary module is used to enrich meal-anchored behavioural episodes rather than to perform clinical dietary assessment. These findings support the feasibility of integrating meal-image interpretation with event-driven motion sensing for context-aware home monitoring.

## 5. Discussion

The experimental results provide initial system-level evidence that the proposed framework can operate in a real kitchen–dining environment and generate structured multimodal behavioural records from irregular domestic activity. Importantly, the present study should be interpreted as a feasibility evaluation of an event-driven sensing and computing framework, rather than as a clinical validation study or a benchmark of a specific pose-estimation or dietary-assessment algorithm. Unlike laboratory-based motion analysis systems that typically rely on predefined tasks, fixed protocols, or continuous recording, the proposed framework focuses on runtime event orchestration, cross-modal temporal organization, privacy-aware local data representation, and practical deployment in a naturalistic home setting. This section discusses the implications of the results for multimodal sensing and computing, the value of event-driven monitoring, the potential use of structured behavioural records for future digital biomarker research, and the remaining limitations that should be addressed before broader clinical or long-term care applications.

### 5.1. Implications for Multimodal Sensing and Computing

The main implication of this study is that multimodal sensing for home monitoring should be considered not only as a problem of combining multiple sensors, but also as a problem of organizing heterogeneous event streams into interpretable behavioural records. In the proposed framework, stereo vision-based motion sensing, dining-zone-triggered meal image acquisition, timestamp-based synchronization, structured local storage, and LLM-assisted dietary context interpretation are integrated into a unified sensing and computing pipeline. This design shifts the focus from isolated sensor outputs to episode-level multimodal representation.

The experimental results support this design direction. During the real-time deployment phase, the system generated 26 event-driven motion sessions, identified 85 presence intervals, and produced 51,165 captured pose frames. These results show that the system was able to transform naturally occurring and fragmented domestic activity into bounded session-level records. This is an important requirement for real-world multimodal sensing because domestic behaviour does not occur as a sequence of clean experimental trials. Instead, it is irregular, intermittent, and context-dependent. A second implication is that event-driven orchestration can improve the practical value of multimodal data. The results showed that sustained active sessions accounted for only 30.8% of all recorded motion sessions but contributed 81.5% of the captured pose frames. In contrast, brief triggers accounted for 42.3% of sessions but contributed only 1.7% of captured pose frames. This uneven distribution indicates that the runtime mechanism was able to preserve high-value activity periods while limiting the relative contribution of low-information transient events. Therefore, event-driven session control can function as a data-structuring mechanism, not merely as a recording trigger. A third implication concerns cross-modal temporal organization. The overlap analysis showed that seven of eight meal-related event records were temporally associated with motion monitoring sessions. This result suggests that meal events can serve as meaningful contextual anchors for organizing movement observations. In conventional multimodal sensing systems, different sensor streams are often synchronized at the frame level or treated as parallel time series. However, in home monitoring, strict frame-level synchronization is not always necessary or practical. The present framework demonstrates that event-level temporal association can provide a more flexible and interpretable structure for daily-life monitoring.

From a computing perspective, the proposed framework also shows the importance of intermediate structured representations. Instead of storing continuous raw video streams, the system retained pose trajectories, timestamps, session metadata, dietary-event records, and synchronized episode information. This representation reduces storage burden, improves data accessibility, and supports later analysis without requiring repeated inspection of raw visual archives. The observed structured pose storage rate of approximately 550 kB/min further suggests that skeletal data can provide a compact motion representation for longer-term monitoring. The integration of LLM-assisted dietary interpretation further extends the role of multimodal computing. In this study, the LLM module was not used to make clinical nutritional judgments. Instead, it was used to convert representative meal images into structured contextual descriptors. Although the observed MAPE of 25.44% indicates that the module is not sufficient for precise nutritional quantification, it can still provide coarse semantic context for meal-anchored behavioural episodes. This suggests that LLM-based interpretation may be useful as a contextual computing layer when its outputs are used conservatively and explicitly separated from clinical measurement.

### 5.2. Value of Event-Driven Monitoring in Real Homes

The results of this study highlight the practical value of event-driven monitoring in real home environments. Domestic activity is fundamentally different from laboratory activity. In a laboratory setting, participants are usually instructed to perform predefined tasks within a controlled spatial region and time period. In contrast, activity in a real home is spontaneous, intermittent, and strongly affected by the layout of the environment, daily routines, furniture placement, occlusion, and temporary movement in and out of the camera view. Therefore, a home-monitoring system must be able to handle irregular timing and fragmented observations without relying on manual session control. The proposed event-driven mechanism addresses this issue by converting detected human presence and dining-zone occupancy into bounded monitoring sessions. The experimental results showed that the system recorded 26 motion sessions and identified 85 presence intervals during the real-time monitoring period. This indicates that a single recorded session often contained multiple active sub-periods. Such fragmentation is expected in real homes, where a person may briefly enter the kitchen, leave the monitored area, return to the dining table, sit down, stand up, or become partially occluded. Event-driven session control makes it possible to preserve these naturally occurring patterns as structured records rather than treating them as noise.

Another important value of event-driven monitoring is that it reduces the need for continuous raw-video recording. Continuous recording may appear attractive from a data-completeness perspective, but it is often impractical in private living spaces. It increases storage burden, creates large amounts of low-value data, and raises privacy concerns because raw videos may contain identifiable persons, private scenes, and unrelated daily activities. By contrast, the proposed framework retains structured skeletal data, event timestamps, session metadata, meal-related images, and cross-modal episode records. This design enables later behavioural analysis while avoiding unnecessary retention of continuous visual streams. The session-category results further support the value of event-driven monitoring. Brief triggers accounted for 42.3% of all recorded motion sessions but contributed only 1.7% of the captured pose frames. In contrast, sustained active sessions accounted for 30.8% of sessions but contributed 81.5% of the captured pose frames. This result suggests that the system did not simply accumulate data whenever the camera was active. Instead, the event-driven mechanism helped separate short transient activations from sessions that contained more continuous and analyzable motion information. This is particularly useful for long-term monitoring, where the amount of data can become difficult to manage if all detections are processed with equal priority.

The use of debounce and cooldown logic is also important in real homes. Immediate session termination after every missed detection would result in excessive fragmentation, especially when the subject is temporarily occluded, partially outside the camera view, or seated in a difficult posture for pose estimation. On the other hand, excessively long session retention would merge unrelated events and reduce interpretability. The proposed framework balances these two problems by allowing temporary disappearance during the cooldown period while still finalizing sessions when absence persists. This state-based design provides a practical compromise between sensitivity and session-level coherence. Event-driven monitoring also improves the interpretability of multimodal data. In continuous recording, the analyst must later identify which time periods correspond to meaningful activity. In the proposed framework, meaningful periods are already organized as motion sessions, dining events, and synchronized behavioural episodes. This makes the data easier to search, compare, and link across modalities. For example, a meal event can be used as an anchor to retrieve associated motion sessions, representative meal images, and dietary-context descriptors. Such organization is essential for context-aware home monitoring because behaviour becomes more interpretable when it is linked to daily-life events.

From an application perspective, event-driven monitoring may also reduce the burden on users. The system does not require the user to manually start or stop recording. This is important for older adults or individuals who may not be able to operate monitoring devices consistently. The system instead responds to naturally occurring presence and dining-related events. Although further usability evaluation is required, this passive and automatic monitoring style is more compatible with long-term deployment in domestic environments than task-based or manually triggered data collection. However, event-driven monitoring also introduces design challenges. The choice of trigger conditions, presence thresholds, dining-zone definitions, debounce parameters, and cooldown duration directly affects the resulting session structure. If the trigger is too sensitive, the system may generate many low-value brief sessions. If it is too strict, meaningful activity may be missed. Therefore, event-driven home monitoring requires careful parameter design and may need adaptive tuning for different home layouts, camera positions, and user routines. The present study provides an initial implementation and feasibility evaluation, but future deployments should examine how these parameters generalize across environments.

### 5.3. Potential for Digital Behavioural Biomarkers

The proposed framework also has potential implications for the development of digital behavioural biomarkers in home environments. Digital behavioural biomarkers refer to measurable behavioural indicators derived from digital sensing data that may reflect changes in daily function, activity patterns, or health-related behaviour. In the context of this study, the framework does not directly establish validated clinical biomarkers. Instead, it provides a sensing and computing foundation for generating structured behavioural records from which future biomarkers may be derived. A key requirement for digital behavioural biomarkers is that data should be collected in ecologically valid settings. Short-term laboratory assessments can provide controlled measurements, but they may not capture how a person actually moves and behaves in daily life. For older adults and people with functional decline, important behavioural changes may appear gradually in ordinary routines, such as reduced movement around meals, longer transition time, unstable standing behaviour, decreased activity continuity, or changes in meal-related routines. These subtle changes are difficult to observe through occasional assessments alone. The proposed framework addresses this problem by capturing daily activity in a real kitchen–dining environment and organizing it into event-driven motion sessions and meal-anchored behavioural episodes. The session-level results suggest several possible behavioural indicators that could be explored in future work. For example, the number of motion sessions, the number of presence intervals, Active Presence Duration, session fragmentation, valid-frame continuity, and the proportion of sustained active sessions may reflect different aspects of daily activity structure. A person who becomes less active or more unstable may show fewer sustained sessions, shorter active presence duration, more fragmented presence intervals, or reduced continuity of valid pose data. Although the present study does not link these measures to health outcomes, it demonstrates that the proposed framework can generate the type of structured data needed to investigate such relationships.

The pose-based outputs also provide a basis for extracting motion-related digital indicators. Three-dimensional skeletal trajectories can be used to compute body displacement, posture variation, trunk movement, standing and sitting transitions, reaching patterns, walking-like movement within the monitored area, and temporal stability of movement. Because the data are organized by session, these features can be calculated at the session level rather than only at the frame level. This is important for behavioural biomarker development because clinically meaningful changes are often expressed as patterns over time, not as isolated poses. Meal-anchored episodes may provide another important direction for behavioural biomarker development. Eating and dining-related routines are meaningful daily-life contexts that often involve multiple functional components, including approaching the dining area, sitting down, reaching, handling objects, standing up, and post-meal movement. By associating meal events with motion sessions, the proposed framework enables the analysis of behaviour around meals. Future indicators may include movement duration before and after meals, frequency of posture transitions during dining-related periods, activity continuity around the dining table, or changes in the temporal relationship between meal events and movement sessions. The integration of dietary-context descriptors may further enrich these behavioural records. In the present study, LLM-assisted dietary interpretation was treated as a coarse contextual layer rather than as a precise nutritional measurement. Accordingly, the LLM-assisted dietary module should be interpreted as a preliminary semantic annotation component for contextualizing behavioural records, rather than as a validated tool for nutritional quantification, dietary diagnosis, or clinical intervention. Nevertheless, even coarse information about meal-related context can help organize and interpret motion data. For example, a motion session associated with a meal event may be interpreted differently from a motion session that occurs without any dining context. This context-aware interpretation is important because behavioural meaning depends not only on movement itself, but also on the activity situation in which the movement occurs.

Another advantage of the proposed framework is that it supports longitudinal data organization. Digital behavioural biomarkers require repeated observations over time. The structured storage of session identifiers, timestamps, pose trajectories, dining-event metadata, and synchronized episode records makes it possible to compare behavioural patterns across days, weeks, or longer periods. For example, future studies could examine whether sustained active session duration decreases over time, whether meal-related movement becomes more fragmented, or whether daily activity patterns become less regular. Such longitudinal changes may be more informative than single-session measurements. However, several steps are necessary before the outputs of the proposed framework can be interpreted as validated digital behavioural biomarkers. First, larger datasets are needed across multiple homes, users, and daily routines. Second, the extracted behavioural features should be compared to established health, functional, or cognitive assessments. Third, the reliability and reproducibility of the features should be evaluated under different camera placements, lighting conditions, occlusion patterns, and household layouts. Fourth, clinically meaningful thresholds or change patterns should be established through collaboration with healthcare professionals. It is also important to avoid overinterpreting sensor-derived features. A change in session duration or pose-frame continuity may reflect health-related change, but it may also be caused by environmental changes, user schedule variation, camera occlusion, or sensor placement. Therefore, behavioural biomarkers should be developed with careful validation and contextual interpretation. The proposed framework contributes to this process by preserving contextual metadata and by organizing data into interpretable event-level records, which can help distinguish behavioural change from technical or environmental variation.

### 5.4. Privacy, Deployability, and Scalability

Privacy, deployability, and scalability are critical considerations for home monitoring systems. Unlike laboratory sensing systems, which are operated in controlled spaces for limited periods, home monitoring systems are expected to operate in private living environments over extended periods. Therefore, the practical value of such systems depends not only on sensing accuracy, but also on whether they can minimize privacy exposure, operate with accessible hardware, and be adapted to different home layouts and daily routines.

#### 5.4.1. Privacy-Aware Data Handling

The proposed framework was designed with privacy-aware data handling as a central requirement. During routine real-time monitoring, the system did not retain continuous raw video streams. Instead, it stored structured outputs, including pose trajectories, timestamps, session metadata, dining-event records, selected meal-related images, and synchronized episode information. This strategy reduces the amount of identifiable visual information retained from the home environment while preserving data that are necessary for behavioural analysis. The use of skeletal pose trajectories is particularly important from a privacy perspective. Raw video frames contain detailed appearance information, such as facial features, clothing, background objects, and private domestic scenes. In contrast, pose trajectories represent the body as a set of keypoint coordinates. Although skeletal data may still reveal behavioural patterns and should not be treated as completely anonymous, they expose less visual detail than raw video. Therefore, structured pose storage provides a practical compromise between data utility and privacy protection. Meal-related image acquisition requires additional care because food images may also capture tableware, room layout, personal objects, or partial human appearance. In the proposed framework, meal images were acquired only when dining-zone events were detected, rather than through continuous image capture. This event-driven acquisition reduces unnecessary visual recording. Nevertheless, future deployments should further strengthen privacy protection through image anonymization, local access control, encryption, user-configurable retention policies, and explicit consent mechanisms. It is also important to distinguish privacy-aware operation from complete privacy preservation. The present implementation reduces privacy exposure by avoiding continuous raw-video retention and by using structured local storage. However, it does not eliminate all privacy risks. Motion trajectories, timestamps, and meal-related records can still reveal sensitive behavioural routines. Therefore, future systems should combine technical minimization with governance mechanisms, including clear data-use policies, secure storage, user review interfaces, and deletion controls.

#### 5.4.2. Deployability in Real Home Environments

The experimental deployment suggests that the proposed framework can be implemented using accessible hardware in a real kitchen–dining environment. The prototype operated on a general-purpose laptop computer without a dedicated high-performance GPU. The sensing setup consisted of a fixed stereo RGB camera for body-motion monitoring and an RGB dietary camera for meal-related image acquisition. This configuration is more practical than systems that require specialized motion-capture equipment, wearable sensors, or laboratory-scale infrastructure.

The use of fixed cameras also reduces the burden on users. Wearable devices require charging, wearing compliance, and user acceptance. In contrast, a fixed environmental sensing system can operate passively after installation. This is especially relevant for older adults or individuals who may not consistently operate devices by themselves. The proposed event-driven design further supports deployability because users do not need to manually start or stop recording sessions.

However, deployability in real homes remains challenging. Camera placement strongly affects the quality of person detection, pose reconstruction, and dining-zone monitoring. Occlusion caused by furniture, table edges, kitchen counters, or body posture can reduce pose quality. Lighting variation may also affect RGB-based sensing, especially during evening or low-light conditions. In addition, the optimal dining-zone definition may differ depending on table shape, room layout, and camera angle. These factors suggest that future deployment tools should include simple calibration procedures, visual setup guidance, and automatic quality checks.

Another practical issue is the balance between system sensitivity and false activation. If detection thresholds are too low, the system may generate many brief triggers or low-value event records. If thresholds are too high, meaningful activity may be missed. The present framework addresses this issue through debounce and cooldown logic, but future systems may require adaptive parameter tuning based on environmental conditions and user routines. Such adaptation will be important for reducing manual configuration effort when deploying the system across different homes.

#### 5.4.3. Scalability of the Framework

The proposed framework is scalable at the data-structure level because it organizes observations into sessions and episodes. Each motion session, dining event, and synchronized behavioural episode is stored with identifiers, timestamps, metadata, and associated file paths. This structure allows additional data to be accumulated over time without relying on continuous video archives. It also enables later filtering by date, event type, session category, overlap status, or behavioural context. The structured storage rate observed in the evaluation also supports scalability. Structured pose outputs required approximately 550 kB per minute of recorded pose data. This compact representation makes it feasible to retain long-term skeletal motion records, especially when combined with event-driven activation. Because the system records data only during behaviourally relevant events, storage growth is more manageable than in continuous recording systems. At the system level, scalability can be considered in three directions. The first is temporal scalability, which refers to longer deployment periods. The present study evaluated a short real-time monitoring period, but the same session-based structure can be extended to weeks or months of monitoring. The second is environmental scalability, which refers to deployment in different homes or care settings. This will require robust calibration, flexible zone definition, and adaptation to different layouts. The third is modal scalability, which refers to adding new sensing modalities, such as depth cameras, pressure sensors, ambient sensors, wearable devices, or smart appliance logs. Because the framework is based on timestamped event records, additional modalities can be integrated as new event streams if their temporal metadata are available. Nevertheless, scalability also introduces new challenges. Long-term monitoring requires reliable device maintenance, automatic recovery from software errors, storage management, and user-friendly configuration. Multi-home deployment requires standardization of data formats, quality-control procedures, and privacy policies. Multi-modal expansion requires careful synchronization and interpretation so that additional sensors improve contextual understanding rather than simply increasing data complexity.

#### 5.4.4. Balancing Data Utility and Practical Constraints

A key design principle of the proposed framework is to balance data utility with practical constraints. High-resolution continuous video may provide rich information, but it is difficult to justify in private homes because of privacy and storage concerns. Minimal sensing may reduce privacy exposure, but it may not provide enough information for behavioural analysis. The proposed framework takes an intermediate approach by using event-driven activation, structured pose representation, selective meal-image acquisition, and local metadata organization. This balance is particularly important for healthcare-oriented home monitoring. Systems intended for daily-life assessment must be acceptable to users, manageable for researchers or care providers, and robust enough for non-laboratory conditions. The present results suggest that event-driven multimodal sensing can provide a practical foundation for such systems. However, broader validation is still necessary before the framework can be used in clinical or large-scale care applications. Future work should therefore evaluate privacy perception, user acceptance, installation burden, and long-term stability in addition to technical performance. For example, user studies may examine whether residents feel comfortable with fixed cameras when continuous raw video is not retained, whether they understand what types of data are stored, and whether they can control or review the retained records. Such human-centered evaluation will be essential for moving from prototype deployment to practical home-monitoring systems.

#### 5.4.5. Summary of Privacy, Deployability, and Scalability

The proposed framework addresses privacy, deployability, and scalability by combining event-driven sensing, structured local storage, and compact pose-based representation. The absence of continuous raw-video retention during routine monitoring reduces privacy exposure, while the use of a general-purpose laptop and fixed RGB cameras supports practical deployment. The session- and episode-based data structure provides a scalable basis for long-term and multimodal extension. At the same time, further work is required to strengthen data security, support adaptive calibration, evaluate user acceptance, and validate the framework across diverse home environments.

### 5.5. Limitations and Future Work

Although the present study demonstrates the feasibility of the proposed event-driven multimodal sensing and computing framework, several limitations should be acknowledged. These limitations are related to the deployment scale, evaluation duration, sensing robustness, dietary interpretation accuracy, clinical validation, and long-term privacy and usability requirements. Addressing these issues will be important for extending the proposed framework from a prototype system to a more generalizable home-monitoring platform. A central limitation is that the current validation remains feasibility-oriented and descriptive. Although the study reports quantitative indicators for session generation, pose-frame usability, motion–meal overlap, storage cost, and dietary-context estimation error, it does not yet provide full component-level validation against external reference systems. Future work should therefore evaluate person-detection accuracy, dining-zone event detection accuracy, synchronization error, processing latency, resource consumption, and pose-reconstruction accuracy under controlled and multi-home conditions.

#### 5.5.1. Limited Deployment Environment

The system was evaluated in a single kitchen–dining environment. This deployment setting was appropriate for verifying whether the proposed framework could operate under naturalistic domestic conditions, but it does not fully represent the diversity of real homes. Different homes may have different room layouts, table positions, lighting conditions, furniture arrangements, camera installation constraints, and activity routines. These factors can affect person detection, stereo pose reconstruction, dining-zone detection, and motion–meal synchronization. Therefore, future work should evaluate the framework across multiple homes and care environments. Such evaluation should include different kitchen and dining layouts, camera positions, table shapes, room sizes, and lighting conditions. Multi-site deployment would make it possible to examine whether the proposed event-driven logic and cross-modal synchronization strategy remain robust under diverse physical conditions. Such multi-home evaluation would also make it possible to determine whether the proposed thresholds and runtime parameters can be generalized or whether adaptive calibration is required for each home environment.

#### 5.5.2. Short Real-Time Monitoring Period

The real-time monitoring evaluation was limited to a short deployment period. Although the system was deployed over 11 days, the evaluation focused on the stabilized real-time monitoring phase from 11 February to 14 February 2026. This period was sufficient to assess the basic feasibility of session generation, pose-frame collection, meal-event anchoring, and structured storage. However, it is not sufficient to evaluate long-term stability, seasonal variation, changes in daily routines, device maintenance requirements, or user adaptation over time. Future work should conduct longer-term monitoring over several weeks or months. Longer deployments would allow the analysis of daily and weekly behavioural patterns, system failure rates, data accumulation speed, calibration drift, and user acceptance. Longitudinal deployment is also necessary for investigating whether the generated motion and meal-anchored behavioural records can support digital behavioural biomarker development. Longer monitoring would also allow the stability of event triggering, storage growth, synchronization performance, and dietary-event coverage to be assessed over time.

#### 5.5.3. Single-User and Limited Activity Scenarios

The current prototype was evaluated under a limited set of real-home activity conditions. The analysis focused mainly on motion activity and meal-related events in a kitchen–dining space.

The current implementation maintains a single global presence and session state and does not assign persistent identities to multiple people detected simultaneously. Consequently, when several residents occupy the monitored area, their detections and trajectories may be merged into the same session, and the association between an individual motion trajectory and a dining event may become ambiguous.

More complex home situations, such as multiple residents, visitors, pets, overlapping activities, or simultaneous use of the kitchen and dining area by multiple people, were not systematically examined. This limitation is important because real homes often include multi-person interactions and ambiguous activity contexts.

Future extensions should first integrate a multi-object tracking (MOT) framework to assign and maintain a temporary track identifier for each detected person across consecutive frames. A person re-identification (Re-ID) module could then reconnect tracks after temporary occlusion, exit, or re-entry, thereby reducing identity switches and preventing observations from different residents from being merged into the same behavioural session. The current global finite-state session controller would also need to be extended to an identity-aware set of per-person state machines, so that presence detection, cooldown handling, session continuation, and session finalization are managed independently for each tracked individual. Dining-zone occupancy should similarly be evaluated for each person track, allowing motion trajectories, dining events, and meal-related records to be associated with the corresponding pseudonymous track identifier. When multiple residents share the same dining event or the available meal images cannot be assigned reliably to one individual, the system should preserve the record as a shared or ambiguous multi-person dining episode rather than forcing a single-person association. Future multi-resident evaluations should quantify identity switches, track fragmentation, person-specific session-generation accuracy, and the correctness of trajectory-to-dining-event associations.

In addition, future studies should evaluate other daily-life contexts beyond dining, such as medication-taking, cooking, cleaning, rehabilitation exercises, and transitions between rooms.

#### 5.5.4. Pose Estimation and Stereo Reconstruction Robustness

The quality of pose estimation and stereo reconstruction may be affected by several practical factors. These include occlusion by furniture, partial body visibility, seated posture, clothing, low illumination, motion blur, camera angle, and limited baseline between stereo cameras. In the present evaluation, valid pose-frame distribution and session classification were used to assess the usability of generated motion data. However, the study did not perform a detailed ground-truth validation of three-dimensional joint accuracy against a reference motion-capture system. Future work should include more rigorous pose-quality evaluation. This may involve comparison with marker-based motion capture, depth-camera measurements, manually annotated keypoints, or wearable inertial sensors. Such validation would help quantify reconstruction error, joint-level reliability, and the conditions under which the system produces stable motion trajectories. In addition, adaptive camera placement guidance and automatic calibration-quality assessment should be developed to support practical deployment by non-expert users.

The current prototype does not include a dedicated occlusion-aware pose-recovery model that can reliably reconstruct severely or persistently occluded lower-limb postures. Kalman filtering and short-term state prediction represent practical temporal-processing approaches for reducing frame-to-frame jitter and bridging brief gaps in keypoint observations. However, predicted coordinates should not be interpreted as reliable recovery of the true limb posture during prolonged or complete occlusion. Future extensions will therefore investigate dedicated occlusion-handling algorithms and temporal pose-tracking models that exploit information from preceding and subsequent frames to estimate temporarily missing lower-limb keypoints. Confidence-aware short-gap prediction, interpolation, skeletal bone-length constraints, joint-kinematic constraints, and uncertainty-based rejection should also be considered to prevent unreliable reconstructed poses from being used directly in downstream behavioural analysis. The effectiveness of these methods should be evaluated under controlled lower-body occlusion conditions using external reference measurements rather than solely through internal skeletal-stability indicators.

#### 5.5.5. Preliminary Dietary Context Estimation

The dietary context estimation module should be regarded as preliminary. The LLM-assisted analysis achieved a MAPE of 25.44% against reference nutrition labels, indicating that it can provide coarse dietary-context descriptions but is not sufficiently accurate for precise nutritional quantification. The result is useful for context-aware episode construction, but it should not be interpreted as a validated dietary assessment method. Future work should improve dietary interpretation by incorporating larger meal-image datasets, controlled reference measurements, multi-angle image capture, food-segmentation methods, portion-size estimation, and user confirmation. Combining image-based interpretation with additional information, such as recipe data, food-weight records, smart kitchen devices, or self-reported intake, may also improve reliability. Importantly, future studies should clearly distinguish between coarse meal-context description and clinically meaningful dietary assessment.

#### 5.5.6. Lack of Clinical and Health-Outcome Validation

The present study did not validate the generated behavioural records against clinical, functional, or cognitive outcomes. The framework provides a data infrastructure for future digital behavioural biomarker research, but it does not yet establish clinically validated biomarkers. For example, the number of sustained active sessions, active presence duration, session fragmentation, valid-frame continuity, or meal-related movement patterns may potentially reflect daily function, but their relationship to health status remains to be examined. Future work should therefore collaborate with healthcare professionals to compare sensor-derived features with established assessment instruments. Depending on the target population, these may include physical function tests, frailty assessments, cognitive screening measures, activities-of-daily-living scales, nutritional assessments, or clinical interviews. Such validation is essential before the framework can be used for health monitoring, early risk detection, or intervention evaluation.

#### 5.5.7. Privacy, Security, and User Acceptance

Although the system was designed to reduce privacy exposure by avoiding continuous raw-video retention during routine monitoring, further privacy and security mechanisms are needed. Pose trajectories, timestamps, meal images, and episode metadata can still reveal sensitive daily routines. Therefore, privacy-aware storage should be strengthened through encryption, access control, data minimization policies, anonymization procedures, user-configurable retention periods, and secure deletion functions. User acceptance should also be evaluated more systematically. Even if raw video is not continuously retained, residents may still feel uncomfortable with fixed cameras in private spaces. Future studies should investigate how users perceive the system, whether they understand what data are stored, whether they can review or delete records, and what types of feedback or control interfaces improve trust. Such human-centered evaluation will be essential for practical deployment in homes and care settings.

#### 5.5.8. Future System Extensions

Several technical extensions are planned. First, the framework can be expanded to include additional sensing modalities, such as depth cameras, thermal sensors, pressure mats, inertial sensors, smart appliance logs, or environmental sensors. Because the current framework is based on timestamped event records, these additional modalities can be incorporated as new event streams. Second, adaptive event-triggering mechanisms should be developed. Instead of using fixed thresholds for presence detection, dining-zone overlap, debounce control, and cooldown duration, future systems may learn personalized parameters based on the user’s routine and the deployment environment. This could reduce false triggers and improve the relevance of recorded sessions. Third, the episode-level data representation can be extended to support automatic feature extraction and anomaly detection. For example, the system could compute daily summaries of activity continuity, meal-related movement duration, posture transition frequency, or changes in routine regularity. These features could then be used for long-term monitoring, caregiver feedback, or research on digital behavioural biomarkers.

## 6. Conclusions

This study proposed an event-driven multimodal sensing and computing framework for context-aware home monitoring using stereo vision and dietary event anchoring. The framework addresses a practical system-design problem in real-world domestic monitoring: daily behaviour is irregular, temporally fragmented, context-dependent, and privacy-sensitive. Rather than relying on continuous raw-video recording or isolated activity classification, the proposed framework coordinates sensing activation, runtime session control, cross-modal synchronization, structured local storage, and coarse dietary-context interpretation to organize daily-life observations into session-level and episode-level behavioural records. The proposed prototype integrates stereo RGB-based three-dimensional human motion sensing, dining-zone-triggered meal image acquisition, finite-state event control, debounce and cooldown logic, timestamp-based motion–meal synchronization, privacy-aware local data representation, and LLM-assisted dietary context interpretation. Through this sensing–computing pipeline, heterogeneous observations from motion and meal-related events can be activated, bounded, synchronized, and stored as interpretable multimodal records suitable for subsequent analysis.

The real-world deployment in a kitchen–dining environment supported the feasibility of the proposed framework. During the stabilized real-time monitoring phase, the system generated 26 event-driven motion sessions and identified 85 presence intervals, indicating that fragmented domestic activity could be converted into bounded monitoring records. Sustained active sessions accounted for 30.8% of all motion sessions but contributed 81.5% of the captured pose frames, suggesting that event-driven orchestration concentrated most motion data within behaviourally meaningful activity windows while limiting the contribution of brief transient triggers. The cross-modal analysis further showed that seven of eight meal-related event records overlapped with motion monitoring sessions, corresponding to an overlap coverage of 87.5%. This result supports the practical use of meal-related events as contextual anchors for organizing surrounding motion observations. By linking motion sessions with dietary-event records through timestamps, the framework can construct synchronized behavioural episodes that are more interpretable than isolated pose trajectories or standalone meal images.

The evaluation also indicated that the proposed storage strategy is suitable for privacy-aware local monitoring. Structured pose outputs required approximately 550 kB per minute of recorded pose data, corresponding to about 33 MB per hour, while continuous raw video streams were not retained during routine real-time monitoring. The prototype operated on a general-purpose laptop computer without requiring a dedicated high-performance GPU. These results suggest that local event-driven sensing, structured storage, and multimodal episode construction can be implemented with accessible computing resources. The LLM-assisted dietary context module achieved a mean absolute percentage error of 25.44% against reference nutrition labels. This error level indicates that the output should not be used for precise nutrient estimation, individual dietary diagnosis, or clinical decision-making. In the proposed framework, the value of the LLM-assisted module lies in providing a coarse semantic description of the meal context, such as the presence of staple foods and side dishes, which can be linked with surrounding motion records. The result therefore supports the use of meal-image interpretation as a contextual computing component, while also emphasizing the need for caution when interpreting numerical dietary estimates.

Several limitations remain. The evaluation was conducted in a single kitchen–dining environment and focused on a short stabilized real-time monitoring period. The system has not yet been validated across multiple homes, diverse layouts, lighting conditions, camera placements, or multi-person scenarios. The reconstructed pose trajectories were not compared to a clinical-grade motion-capture reference system, and the generated behavioural records were not validated against clinical, functional, cognitive, or nutritional outcomes. Therefore, the present study should be regarded as a system-level feasibility evaluation of a multimodal sensing and computing framework, not as evidence of clinical effectiveness. Future work will extend the framework through longer-term and multi-home deployments, external validation of pose reconstruction accuracy, improved robustness under occlusion and lighting variation, more reliable dietary-context interpretation, adaptive event-threshold tuning, and support for additional sensing modalities. Collaboration with healthcare professionals will also be needed to examine whether session-level motion features, meal-related movement patterns, and longitudinal behavioural changes can be associated with validated health or functional measures. Overall, the proposed framework provides a practical sensing and computing foundation for transforming irregular domestic observations into structured, temporally indexed, and privacy-aware multimodal behavioural records for future home monitoring applications.

## Figures and Tables

**Table 1:** Comparison of representative related work and the present study for home monitoring.

Study	Main Focus	Event and Context Handling	Difference from Our Study
Ghorbani et al. [30]	IoT-based in-home monitoring of older adults	Uses IoT-embedded devices and decision-aware monitoring for assisted living.	Does not integrate stereo motion sensing, meal-event anchoring, or motion–dietary synchronization.
Srivatsa and Plötz [35]	Sensor-based smart home human activity recognition	Models relationships among ambient sensor activations using graph-based representations.	Focuses on activity recognition from sensor events, rather than multimodal behavioural episode construction.
Yan et al. [43]	Nutrition estimation from food images	Uses multimodal large language models and retrieval-augmented generation for dietary estimation.	Focuses on nutrition estimation, whereas our study uses dietary information as contextual anchors for motion monitoring.
Our study	Context-aware home monitoring around motion and meals	Uses event-driven session control, dining-zone triggering, debounce and cooldown logic, and motion–meal temporal synchronization.	Constructs structured, privacy-aware, meal-anchored multimodal behavioural records from irregular domestic observations.

**Table 2:** Hardware and camera specifications of the experimental setup.

Component	Specification
Host computer	Processor: Intel(R) Core(TM) Ultra 5 125U; memory: 16.0 GB RAM; graphics: integrated Intel(R) Graphics; storage: 477 GB SSD; operating system: 64-bit Windows environment.
Body-motion camera	Dual-lens stereo RGB camera; composite acquisition resolution of 3200 × 1200 pixels at a configured camera input rate of 60 fps; each composite frame was horizontally divided into synchronized left and right views of 1600 × 1200 pixels. The left view was used for lightweight person detection and presence gating, while both views were used for two-dimensional pose estimation and stereo triangulation.
Dietary camera	RGB camera; resolution up to 4K; optical zoom: 16×; focal range: F/3.2–7.36 mm; used for meal-related image acquisition around the dining table.

**Table 3:** Runtime parameters used in the prototype implementation during the real-time monitoring phase.

Parameter	Value	Role in the Framework
Person detection confidence threshold	0.3	Determines whether a detected person is accepted as a valid presence event for activating or maintaining motion monitoring.
Absence threshold	15 detection checks	Defines the number of consecutive missed detections required before absence is confirmed, reducing the influence of transient detection failures.
Cooldown duration for motion sessions	300 s	Keeps a motion session open after confirmed absence to preserve continuity across short departures or occlusions.
Dining-zone overlap threshold	0.5	Determines whether the detected person sufficiently overlaps with the predefined dining region to trigger a dining event.
Meal image acquisition interval	30 s	Controls repeated meal-image capture during an active dining event while limiting redundant image acquisition.
Presence-interval gap threshold	3 s	Separates effective presence intervals during offline analysis when the temporal gap between consecutive pose frames exceeds this threshold.
Minimum valid segment length	30 consecutive valid frames	Defines a continuous valid pose segment for calculating the Valid Segment Ratio (VSR).

**Table 4:** Evaluation dimensions and metrics used to assess the proposed framework.

Evaluation Dimension	Metric	Purpose
Event-driven monitoring effectiveness	Number of sessions, presence intervals, Active Presence Duration (APD), session classification	To examine whether the runtime mechanism can convert irregular domestic activity into bounded monitoring sessions.
Motion data usability	Total pose frames, valid frames, Valid Frame Ratio (VFR), Valid Segment Ratio (VSR)	To evaluate whether the generated skeletal motion streams are sufficiently usable and continuous for downstream behavioural analysis.
Cross-modal synchronization utility	Number of meal sessions, number of motion–meal overlaps, synchronized behavioural episodes, overlap duration	To assess whether meal-related events can serve as contextual anchors for organizing motion and dietary observations.
Runtime and storage feasibility	Structured storage size, storage rate, local computing platform, GPU requirement, raw-video retention status	To examine whether the prototype can support practical local operation and privacy-aware data retention in a home monitoring scenario.
Dietary context interpretation	Mean absolute percentage error (MAPE), item-level estimation error, structured output availability	To evaluate whether meal images can provide coarse dietary-context descriptors for synchronized behavioural episodes.

**Table 5:** Session classification criteria based on active presence duration and tracking quality.

Category	Criteria	Interpretation
Brief trigger	APD<20 s	Short transient activation, likely corresponding to a brief appearance or a non-sustained trigger.
Intermittent session	20 s ≤APD<60 s, or VFR≤0.15	Partial activity episode with fragmented monitoring intervals or unstable pose tracking.
Sustained active session	APD≥60 s and VFR>0.15	Continuous activity episode with sufficiently stable tracking for subsequent behavioural analysis.

**Table 6:** Overview of event-driven motion sessions recorded during the real-time deployment phase.

Metric	Value
Evaluation period	11–14 February 2026
Number of recorded motion sessions	26
Number of detected presence intervals	85
Total captured pose frames	51,165
Valid pose frames	25,925
Overall valid-frame yield	50.7%
Average number of presence intervals per session	3.27

**Table 7:** Session type distribution and frame contribution during the real-time deployment phase.

Session Type	Sessions, *n* (%)	Captured Frames, *n* (%)	Interpretation
Brief trigger	11 (42.3%)	877 (1.7%)	Short transient activations or brief appearances with limited analytical value.
Intermittent session	7 (26.9%)	8605 (16.8%)	Partially usable activity episodes, but fragmented by interruptions or unstable tracking.
Sustained active session	8 (30.8%)	41,683 (81.5%)	Main source of analyzable motion data, indicating that the runtime mechanism concentrated most captured frames into high-value sessions.

**Table 8:** Distribution of captured pose frames across event-driven motion-session categories.

Session Type	Sessions, *n* (%)	Captured Pose Frames, *n* (%)	Mean Captured Frames per Session
Brief trigger	11 (42.3%)	877 (1.7%)	79.7
Intermittent session	7 (26.9%)	8605 (16.8%)	1229.3
Sustained active session	8 (30.8%)	41,683 (81.5%)	5210.4
Total	26 (100.0%)	51,165 (100.0%)	1967.9

**Table 9:** Cross-modal overlap between meal-related events and motion monitoring sessions.

Metric	Value
Number of meal-related event records	8
Meal events with at least one overlapping motion session	7
Meal events without overlapping motion sessions	1
Overlap coverage of meal events	87.5%
Meal-anchored synchronized behavioural episodes	7

**Table 10:** Storage efficiency and runtime feasibility indicators of the proposed prototype.

Indicator	Observation
Local computing device	Intel(R) Core(TM) Ultra 5 125U processor, 16.0 GB RAM, integrated Intel(R) Graphics, and 477 GB SSD.
Dedicated GPU requirement	No dedicated high-performance GPU was used.
Configured stereo-camera input	3200 × 1200 composite frames at 60 fps, divided into synchronized 1600 × 1200 left and right views.
Mean motion-session storage size	1.87 MB per session.
Median motion-session storage size	0.29 MB per session.
Motion-session storage range	0.013–16.85 MB.
Structured pose storage rate	551.9 kB/min; median 465.1 kB/min.
Storage per 1000 pose frames	0.84 MB; median 0.97 MB.
Average CPU utilization during active monitoring	Approximately 77%.
Average memory footprint during active monitoring	Approximately 892 MB.
Configured user-interface update interval	50 ms.
Detection-to-session-start latency	No more than approximately 150 ms on average.
Routine raw-video retention	Continuous raw-video streams were not retained during routine real-time monitoring.
Storage format	CSV files for two-dimensional and three-dimensional pose trajectories; JSON files for session, event, and episode metadata.

**Table 11:** Summary of LLM-assisted dietary context estimation results.

Evaluation Item	Result and Interpretation
Input data	Representative meal images selected from dining-event sessions.
Output format	Structured dietary-context descriptors stored as JSON-style records.
Evaluation reference	Reference nutrition labels for the evaluated meal images.
Main metric	Mean Absolute Percentage Error (MAPE).
Overall MAPE	25.44%.
Interpretation	Suitable for coarse dietary-context description, but not sufficient for precise nutritional quantification.
Role in the proposed framework	Provides contextual descriptors that can be linked with motion sessions to construct synchronized behavioural episodes.

## Data Availability

The data presented in this study are not publicly available due to privacy and ethical restrictions related to home-environment sensing data and clinical research data. De-identified and processed data may be available from the corresponding author upon reasonable request, subject to institutional and ethical restrictions.
